# Measuring nanoscale viscoelastic parameters of cells directly from AFM force-displacement curves

**DOI:** 10.1038/s41598-017-01784-3

**Published:** 2017-05-08

**Authors:** Yuri M. Efremov, Wen-Horng Wang, Shana D. Hardy, Robert L. Geahlen, Arvind Raman

**Affiliations:** 1 School of Mechanical Engineering, Purdue University, West Lafayette Indiana, 47907 USA; 2 Birck Nanotechnology Center, Purdue University, West Lafayette Indiana, 47907 USA; 3 Department of Medicinal Chemistry and Molecular Pharmacology, Purdue University, West Lafayette Indiana, 47907 USA; 4 Purdue University Center for Cancer Research, Purdue University, West Lafayette Indiana, 47907 USA

## Abstract

Force-displacement (*F*-*Z*) curves are the most commonly used Atomic Force Microscopy (AFM) mode to measure the local, nanoscale elastic properties of soft materials like living cells. Yet a theoretical framework has been lacking that allows the post-processing of *F*-*Z* data to extract their *viscoelastic* constitutive parameters. Here, we propose a new method to extract nanoscale viscoelastic properties of soft samples like living cells and hydrogels directly from conventional AFM *F*-*Z* experiments, thereby creating a common platform for the analysis of cell elastic and viscoelastic properties with arbitrary linear constitutive relations. The method based on the elastic-viscoelastic correspondence principle was validated using finite element (FE) simulations and by comparison with the existed AFM techniques on living cells and hydrogels. The method also allows a discrimination of which viscoelastic relaxation model, for example, standard linear solid (SLS) or power-law rheology (PLR), best suits the experimental data. The method was used to extract the viscoelastic properties of benign and cancerous cell lines (NIH 3T3 fibroblasts, NMuMG epithelial, MDA-MB-231 and MCF-7 breast cancer cells). Finally, we studied the changes in viscoelastic properties related to tumorigenesis including TGF-β induced epithelial-to-mesenchymal transition on NMuMG cells and Syk expression induced phenotype changes in MDA-MB-231 cells.

## Introduction

In recent years interest has increased in the measurement of the viscoelastic properties of soft biological samples motivated by their correlation with disease, differentiation, or cellular transformation^1–4^. A large variety of methods have been introduced for measuring cellular mechanical properties including micropipette aspiration^5, 6^, stretching or compression between two microplates^7–9^, optical tweezers^10, 11^, and magnetic twisting cytometry^12, 13^. However, indentation with the atomic force microscope (AFM) remains one of the most popular methods for probing the nanoscale properties of soft samples like cells, tissues and hydrogels^14–17^.

In the AFM, the *elastic* properties of live cells are usually evaluated from force versus displacement (*F*-*Z*) curves. Then the Hertz model or its modifications are applied to the approach part of the *F*-*Z* curve to extract Young’s modulus (*E*_*Hertz*_), the elastic parameter used for characterization of the sample’s mechanical properties^18, 19^. However, such models assume a purely elastic nature of the sample, while in reality most biological samples are viscoelastic. Viscoelasticity is revealed in a clear hysteresis between the approach and retraction parts of curves^20^; the indentation speed dependence of *E*_*Hertz*_ values extracted from force curves with the Hertz model^11^; the observations of force relaxation at constant indentation depth and the creep at constant loading force^21^.

Approaches other than the standard *F*-*Z* curves are usually used to obtain the *viscoelastic* properties of samples with AFM in both the time^22–24^ and frequency domains^25–30^. These generally require modifications in the AFM apparatus and/or in the data acquisition protocol. Equally importantly, each approach has its own sets of measurement uncertainties.

If a standard *F*-*Z* curve could also be used to quantify viscoelastic properties, it would allow one standard method with well quantified uncertainties^31^ to be used for both viscoelasticity and elasticity measurements. This has not been possible to date, we believe, due to the lack of a mathematical/computational framework that allows the post-processing of force-displacement data to extract the relevant viscoelastic constitutive parameters.

Here we propose a new method to extract viscoelastic properties of soft samples like cells and hydrogels directly from standard AFM *F*-*Z* curves. This method is based on the theoretical model developed by Ting^32^ for the problem of the indentation of a linear viscoelastic half-space by a rigid axisymmetric indenter for arbitrary load history. We combine Ting’s model together with related numerical procedures to process the data from both approach and retraction phases of the experimental *F*-*Z* curves. The method is validated with finite element (FE) simulations, with experiments performed on polyacrylamide hydrogel (PAAm) samples, and by comparison with the existed AFM viscoelasticity measurement techniques. The method is then applied to measure the viscoelastic properties of several benign and cancerous cell lines (NIH 3T3, NMuMG, MDA-MB-231, MCF-7), and to study the sensitive changes in viscoelastic properties related to tumorigenesis including TGF-β-induced epithelial-to-mesenchymal transition on NMuMG cells and Syk expression-induced phenotype changes in MDA-MB-231 cells. The method also provides a way to test which constitutive viscoelastic relations best fit the acquired data. We tested applicability of two commonly used relaxation models, standard linear solid (SLS) and power-law rheology (PLR), on data collected on hydrogels and the above cell lines.

We show that the developed method is highly robust, does not require changes in experimental design and is compatible with findings from conventional AFM microrheology. By bringing rigorous viscoelastic analysis within the reach of standard, automated AFM force curves, the method is expected to greatly enhance the wider use and adoption of AFM for living cell and soft biomaterials visco-mechanics assays.

## Results

**Figure 1:**
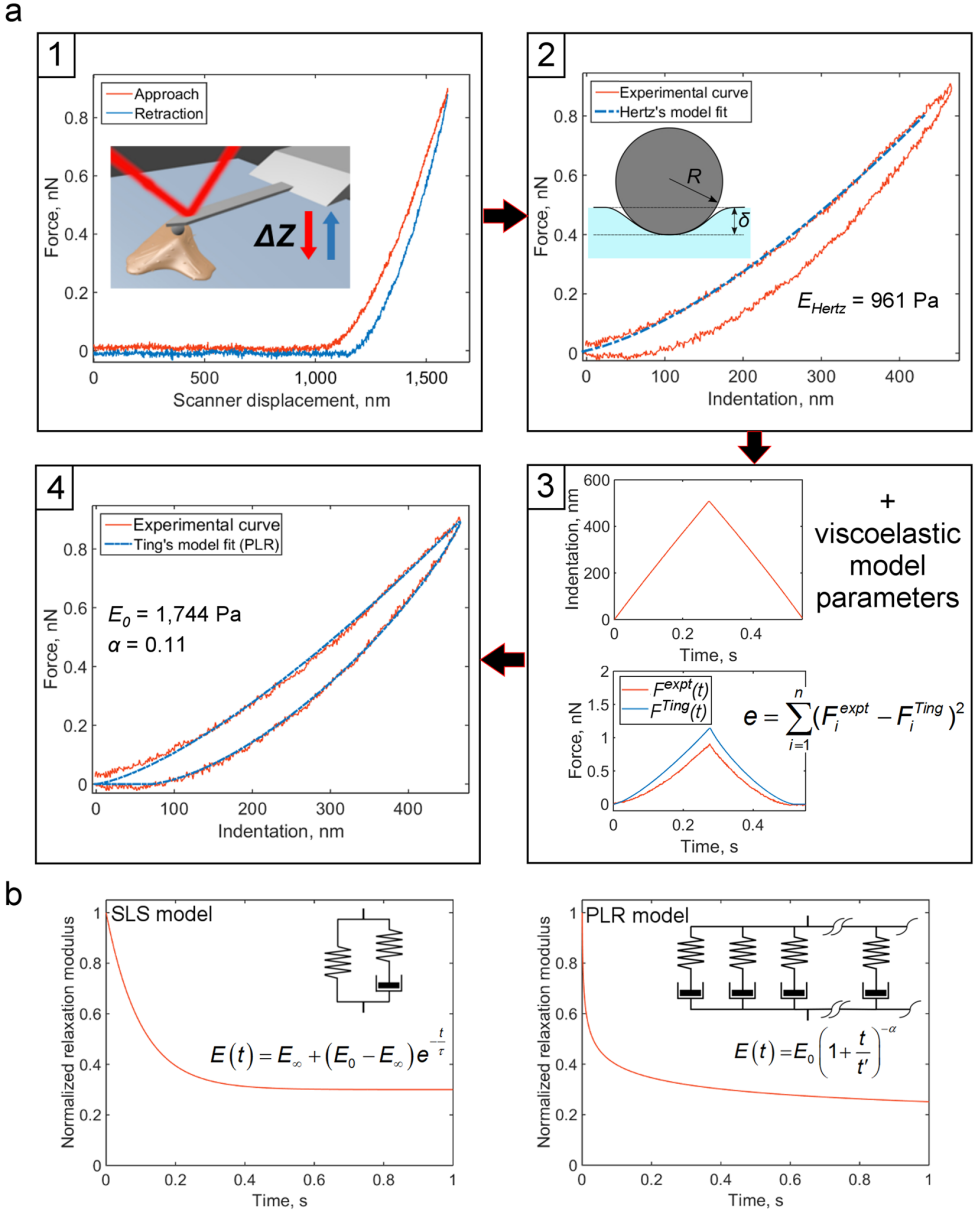
Flowchart of the method and used viscoelastic models. (**a**) The flowchart. At the first step, a force-displacement curve obtained with a standard indentation protocol is pre-processed to determine zero force level and corrected for hydrodynamic drag (if needed). At the second step, the contact point is located and *F*-*Z* curve is transformed to the force-indentation coordinates. Apparent Young’s modulus *E*_*Hertz*_ is calculated with the Hertz’s model. Inset: scheme of a spherical probe indenting a half-space. At the third step, the indentation time history is used to model the force curve with the Ting’s model and prescribed viscoelastic model. The difference between the modelled and experimental force curves (*e*) is minimized with the fitting algorithm. Viscoelastic parameters providing the lowest *e* value are acquired as the output. At the final step, contact point position could be adjusted and the step 3 repeated to obtain the best fit. Details of the processing are given in the text. (**b**) Normalized relaxation modulus (*E*/*E*_0_) for standard linear solid (SLS) and power-law rheology (PLR) relaxation models. Insets: schematic spring-dashpot representation and equation for the relaxation modulus. The SLS model is a spring in parallel with a spring-dashpot combination, leading to an exponential relaxation with a single relaxation time *τ*. The PLR model can be imagined as an infinite number of spring-dashpot combinations in parallel, leading to a continuous relaxation spectrum and power-law decay (*α* is the power law exponent).

Experimental AFM force curves on live cells display viscoelastic behaviour that simply cannot be captured or explained by elastic contact mechanics models. The schematic setup for the method (Fig. 1a) is a commonly used indentation protocol in which the piezo scanner moves the cantilever base relative to the sample with predefined vertical speed profile, which is identical for the approach and retraction phases. The data from such an experiment is the so called force curve (*F*-*Z* curve) representing force vs scanner displacement or, after processing, force vs indentation (*F-δ* curve) dependency. It can be seen from the typical force-indentation curve obtained on a living cell (and also polyacrylamide (PAAm) gels, Fig. S9) that Hertz’s model fits the approach phase fairly well. Not surprisingly, the framework of Hertz contact mechanics^19, 33^ is commonly used for the description of AFM force curves, at least for shallow indentations and low indentation rates^1, 18, 34–37^. However, even at low piezo speeds such as 2 μm/s, viscoelastic behaviour can be observed in the hysteresis between approach and retraction phases, which is not captured by the Hertz’s model (Fig. 1a).

Therefore, to account for the viscoelastic nature of the sample, more advanced tip-sample interaction models are needed. Ting’s model^32^ provides a solution for the problem of indentation of a linear viscoelastic half-space by a rigid axisymmetric indenter for any load history, including approach and retraction phases of *F*-*Z* curves. The following equations describe Ting’s solution for indentation of a viscoelastic sample with a rigid spherical indenter:

1 $$F(t,\delta (t))=\{\begin{array}{cc}\frac{4\sqrt{R}}{3(1-{\nu }^{2})}{\int }_{0}^{t}E(t-\xi )\frac{{\rm{\partial }}{\delta }^{\frac{3}{2}}}{{\rm{\partial }}\xi }d\xi , & 0\le t\le {t}_{m}\\ \frac{4\sqrt{R}}{3(1-{\nu }^{2})}{\int }_{0}^{{t}_{1}(t)}E(t-\xi )\frac{{\rm{\partial }}{\delta }^{\frac{3}{2}}}{{\rm{\partial }}\xi }d\xi , & {t}_{m} < t\le {t}_{ind}\end{array},$$

2 $${\int }_{{t}_{1}(t)}^{t}E(t-\xi )\frac{\partial \delta }{\partial \xi }d\xi =0,$$

where *F* is the force acting on the cantilever tip; *δ* is the indentation depth; *t* is the time initiated at the time of initial contact (*t*_*m*_ is the duration of approach phase; *t*_*ind*_ is the duration of complete indentation cycle); *t*_1_ is the auxiliary function determined by the equation (2); *ξ* is the dummy time variable required for the integration; *E*(*t*) is the Young’s relaxation modulus; *v* is the Poisson’s ratio of the sample (assumed to be time-independent); and *R* is the radius of the indenter. A time-dependent relaxation modulus *E*(*t*) allows for arbitrary linear viscoelastic constitutive equations to be used with Ting’s model. The relaxation modulus is generally a decaying function that can be described by rheological models, like the standard linear solid (SLS) and power-law rheology (PLR) models used here. The corresponding *E*(*t*) functions are:

3 $$E(t)={E}_{\infty }+({E}_{0}-{E}_{\infty }){e}^{-\frac{t}{\tau }},$$

4 $$E(t)={E}_{0}{(1+\frac{t}{t^{\prime} })}^{-\alpha }.$$

The normalized SLS and PLR relaxation functions are presented in Fig. 1b. The SLS model is characterized by three parameters, *E*_0_, *E*_∞_, and *τ* representing the instantaneous and long term elastic modulus and relaxation time, respectively. In the SLS model the extent of relaxation can be determined by the *E*_∞_/*E*_0_ and *τ/t*_*ind*_ (Deborah’s number) ratios. The PLR model is characterized by two parameters, the instantaneous elastic modulus *E*_0_ and the power law exponent *α; t*′ is a small time offset which does not affect relaxation behaviour at experimental times). A larger *α* value means larger relaxation; materials exhibit a solid-like behaviour at *α* = 0, and a fluid-like behaviour at *α* = 1.

We have developed numerical procedures to: first, simulate the *F*-*Z* curves with Ting’s equations (1) and (2) and prescribed viscoelastic relaxation model; and, second, to fit the experimental *F*-*Z* curves to the Ting’s equations to extract the viscoelastic constitutive parameters of the sample for a chosen viscoelastic relaxation model (Fig. 1, Fig. S2). All algorithms were implemented in MATLAB and are described in the Materials and Methods section.

**Figure 2:**
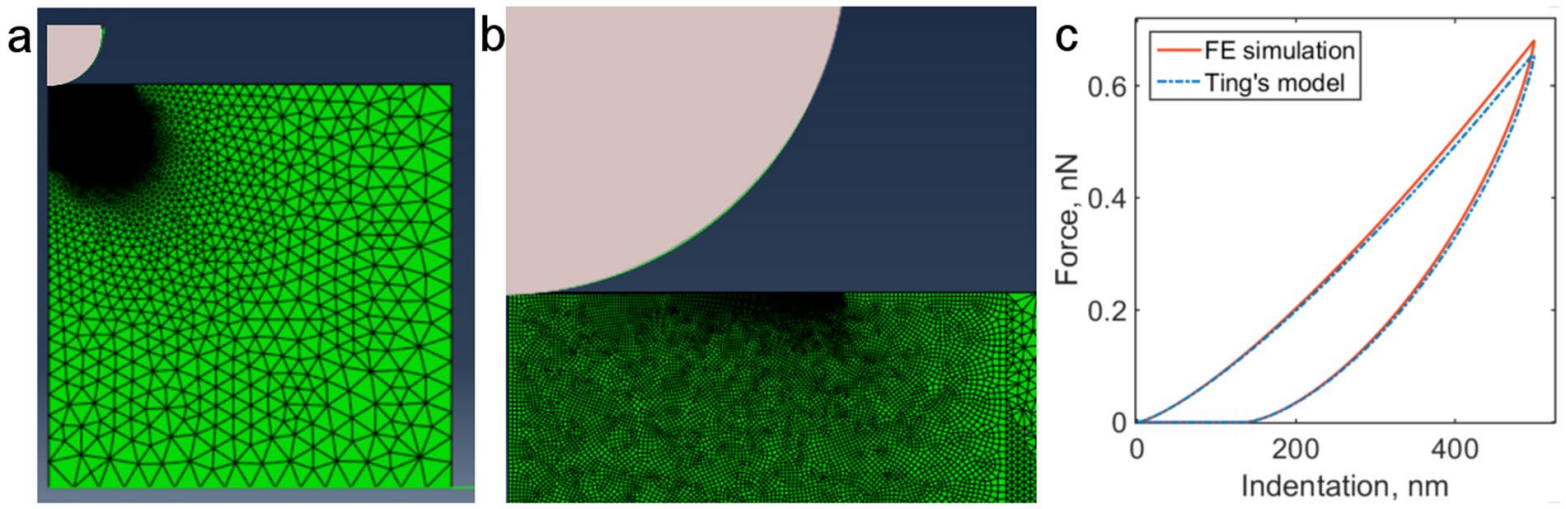
Validation of the developed algorithms with FE simulations. (**a**) Axisymmetric FE model, consisting of the rigid spherical indenter with 2 μm radius and viscoelastic sample with 15 μm height and radius. (**b**) Magnified view of the contact area, there the sample was meshed more finely. (**c**) Result of FE simulation and the *F-δ* curve modelled using Ting’s model with the same input parameters (SLS model with *E*_0_ = 2 kPa, *E*_∞_ = 1 kPa, and *τ* = 0.1 s).

To test the validity of the derived algorithms, we used finite element (FE) analysis. The FE model represented the indentation experiment where the spherical probe indents the sample with predefined viscoelastic constitutive relation (SLS or PLR). The model-predicted (numerically simulated) and FE-simulated curves were markedly similar for the same input parameters (Fig. 2). Some discrepancy appeared at higher depths and may be associated with finite-size effects. Then the fitting procedure was applied to the FE-simulated curves. An excellent agreement between FE-simulated curve and the results of the fitting procedure (R^2^ > 0.99) was observed for both SLS and PLR models; the difference between fitted parameters and parameters used in the FE-model was below 4%. Additional FE simulations to check the effects of finite sample thickness and nonconstant tip velocity that arises from the cantilever deflection are described in the Supplementary Information, Section B.

**Figure 3:**
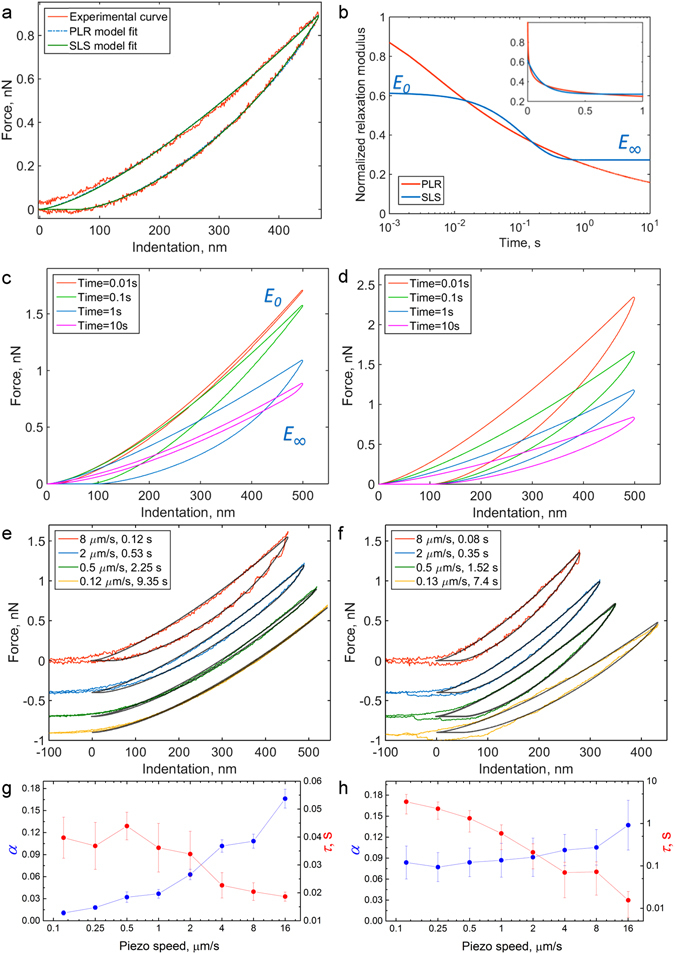
Comparison of SLS and PLR models in experiments with PAAm hydrogels and NIH 3T3 fibroblasts. (**a**) Experimental *F-δ* curve obtained on a fibroblast with both PLR and SLS model fits. (**b**) Normalized relaxation functions for PRL and SLS models with adjusted parameters on a logarithmic scale, inset – same functions on a linear scale. (**c,d**) Modelled *F-δ* curves with different indentation times. Left – SLS (*τ* = 0.1 s), right – PLR (*α* = 0.15). (**e,f**) Experimental *F-δ* curves obtained on PAAm hydrogel (left) and fibroblast (right) with different indentation times. The offset is added to the force for clarity. Black lines are SLS (**e**) or PLR (**f**) model fits. (**g,h**) Viscoelastic parameters *α* and *τ* for PAAm hydrogels (left, combined data for experiments on 3 gels, mean ± s.d.) and NIH 3T3 fibroblasts (right, combined data for experiments on 12 cells, mean ± s.d.) as a function of piezo displacement speed.

Then, we applied the developed method directly to the force curves obtained on living NIH 3T3 fibroblasts and found that both viscoelastic models, SLS and PLR, fit the experimental *F-δ* curve fairly well (Fig. 3a). Indeed, both relaxation functions are quite similar when data spans only a single time decade, but differences between them are expected to become apparent on a logarithmic scale over several time decades (Fig. 3b). The SLS model has plateau regions at both short and long times, corresponding to *E*_0_ and *E*_∞_ modulus, while PLR model does not.

In an effort to distinguish which viscoelastic model best describes the experimental data, numerical simulations using Ting’s model were performed by varying the indentation time *t*_*ind*_ (Fig. 3c and d). As can be seen from the simulated curves using SLS constitutive relations, at both low (≪1) and high (≫1) *τ*/*t*_*ind*_ ratios the approach-retraction hysteresis is small and a complete curve might be described by single modulus *E*_∞_ or *E*_0_ (Fig. 3c). Numerical simulations using the PLR constitutive relation, on the other hand, show that the amount of relaxation varied only slightly (Fig. 3d), the hysteresis area was always present and demonstrated slight growth with *t*_*ind*_. These numerical studies suggest that experimental data should be acquired with different values of *t*_*ind*_ to better distinguish the applicability of a specific viscoelastic constitutive relation.

By varying the piezo displacement speed on NIH 3T3 cells and PAAm hydrogels we found that the PLR constitutive relation better describes the viscoelastic data over a wider range of timescales for live cells while the SLS better described the data for the PAAm hydrogels. Specifically, the piezo scanner displacement speed was varied in a range of 64–16,000 nm/s, which covers the majority of published AFM indentation experiments, thus leading to indentation time in a range of 25 ms–6 s (for a ~500 nm indentation depth). For NIH 3T3 cells, the amount of hysteresis (hysteresis area) in *F-δ* curves was always significant and increased slightly with scanner velocity (more greatly for the highest speed point), indicating prevalence of the PLR model (Fig. 3f and Supplementary Fig. S11). Indeed, after processing with the PLR model, we obtained approximately the same values of power law exponent *α* for all used scanner displacement speeds except the highest (16 μm/s), for which the *α* value increased ~30%. For the same data, the SLS model provided dramatically decreasing (by two orders) relaxation time *τ* values proportionally to the piezo speed, meaning that the results were highly dependent on experimental conditions (Fig. 3h). These results are consistent with the previous observation of power-law decay in step-hold force relaxation experiments^38, 39^ and in microrheology^2, 26, 40^ experiments on cells. In case of the PAAm hydrogels, the hysteresis area almost vanished at indentation times larger than 1 s (piezo speed below 0.5 μm/s); and such force curves might be used for acquisition of *E*_∞_ modulus (Fig. 3e,g and Fig. S11C). At shorter times, the hysteresis area gradually increased. The SLS model provided consistent results for indentation times greater than 0.1 s with *τ* about 0.04 s. At shorter times (higher piezo speeds), *τ* values started to decrease, indicating the presence of several relaxation mechanisms. Application of the PLR model, however, resulted in gradually increased *α* values, indicating its poor applicability for describing PAAm hydrogel relaxation. Since it better describes our live cell data, we used the PLR constitutive relation for processing the rest of the data obtained on living cells.

To compare the presented method with the previous techniques (step-hold stress relaxation and microrheology) the experiments were conducted on PAAm hydrogels (3 samples) and NIH 3T3 cells (40 different cells) at the same locations in the random order. The comparison showed a reasonably good agreement between all three methods, the details are given in the Supplementary Information, Section C.

**Figure 4:**
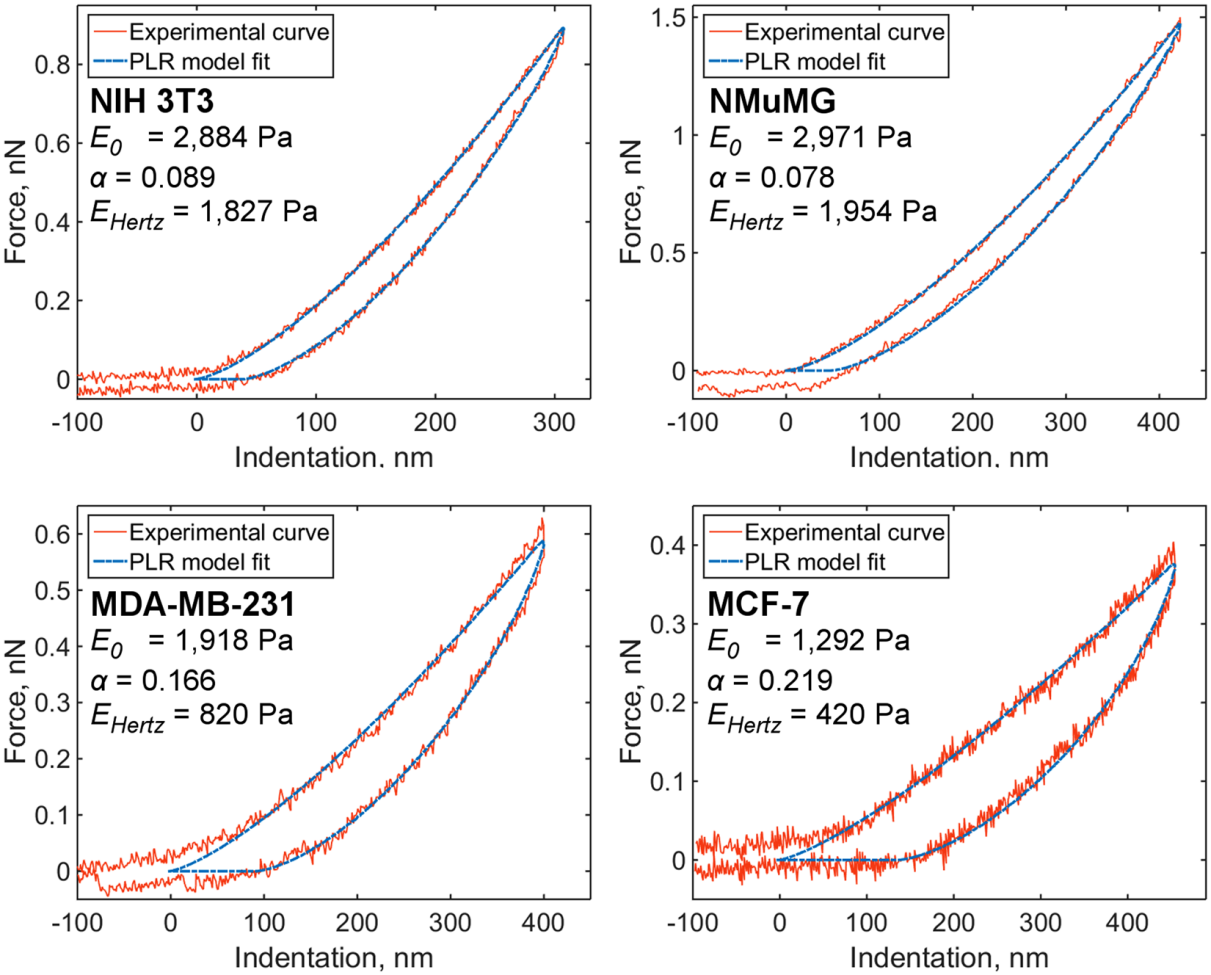
Force curves obtained on different cell lines: NIH 3T3, NMuMG, MDA-MB-231, MCF-7. The fit with PLR model; *E*_*Hertz*_, short-term modulus *E*_0_ and power-law exponent *α* values are shown.

**Figure 5:**
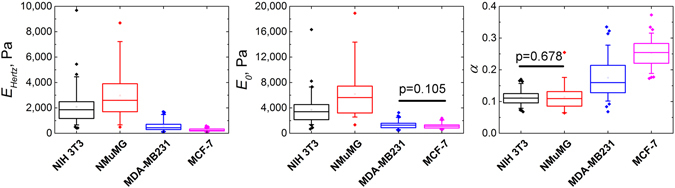
Viscoelastic parameters of studied cell lines. Box plots of apparent Young’s modulus *E*_*Hertz*_, short-term modulus *E*_0_ and power-law exponent *α*. Difference between all distribution except those marked is significant at the p < 0.01 level. All cell lines were at 60–80% confluence during the experiment.

With the described Ting’s model-based method and the PLR relaxation model, we measured the viscoelastic properties of several cell lines: benign murine NIH 3T3 fibroblasts and epithelial NMuMG cells; human breast cancer cell lines MDA-MB-231 and MCF-7. The representative examples of the experimental *F-δ* curves for each cell line are shown in Fig. 4. In agreement with previous work^41^, *E*_0_ values exhibited a log-normal distribution and *α* values were distributed normally (Supplementary Fig. S12). The benign cell lines NIH 3T3 and NMuMG had higher values of *E*_0_ and lower values of *α* than did the cancer cell lines MDA-MB-231 and MCF-7 (p < 0.01 between all parameters except *E*_0_ for pair of cancer cells and *α* for the pair of benign cells), which were both softer and more viscous (Table 1, Fig. 5). The results are in line with the data obtained in previous work^2^, where AFM microrheology experiments were performed in the frequency domain on the same cell lines.

**Table 1:** Viscoelastic parameters of studied cell lines. Mean ± s.d. (median ± m.a.d.).

Cell line	*E*_*Hertz*_ (kPa)	*E*_0_ (kPa)	*α*	*α* data from AFM microrheology experiments* (mean ± S.E.M.)
NIH 3T3 (N = 69)	2.1 ± 1.5 (1.9 ± 0.7)	3.7 ± 2.3 (3.4 ± 1.3)	0.11 ± 0.03 (0.11 ± 0.01)	0.15 ± 0.01
NMuMG (N = 60)	3.0 ± 1.8 (2.6 ± 1.1)	6.2 ± 3.8 (5.6 ± 2.2)	0.11 ± 0.04 (0.11 ± 0.02)	0.16 ± 0.02
MDA-MB-231 (N = 69)	0.55 ± 0.39 (0.44 ± 0.17)	1.3 ± 0.65 (1.3 ± 0.4)	0.17 ± 0.06 (0.16 ± 0.04)	0.22 ± 0.01
MCF-7 (N = 74)	0.26 ± 0.1 (0.23 ± 0.07)	1.2 ± 0.4 (1.1 ± 0.3)	0.25 ± 0.04 (0.25 ± 0.03)	0.25 ± 0.02

N is the number of measured cells. All cell lines were at 60–80% confluence during the experiment. *Data from ref. 2.

It is interesting to check if the correlation exists between extracted viscoelastic parameters. Although cell lines with higher *E*_0_ had lower values of *α*, inside each cell line the parameters *E*_0_ and *α* did not correlate with each other. The apparent Young’s modulus measured with the Hertz’s model *E*_*Hertz*_ demonstrated a weak negative correlation with *α*, which was larger for cancer cells (Pearson’s *r* from −0.1 for NIH 3T3 to −0.6 for MDA-MB-231, p < 0.05). A strong, significant negative correlation was observed between *α* and the *E*_*Hertz*_/*E*_0_ ratio (≈−0.9, p < 0.001), meaning that *E*_*Hertz*_ values depend highly on the amount of relaxation. The example of cancer cells MDA-MB-231 and MCF-7 shows the same effect: both have close *E*_0_ values, but *E*_*Hertz*_ is two times lower for MCF-7 cells due to larger *α* value.

Then we applied the method to study changes in cell mechanical properties in two processes of relevance to cancer tumorigenesis. In the first case study, we compared the difference between control and induced Syk-expressing MDA-MB-231 cells. The Syk protein-tyrosine kinase plays roles in tumour progression, acting as an inhibitor of cellular motility and metastasis in highly invasive cancer cells. Here, both an increase in *E*_0_ and decrease in *α* in Syk-expressing cells was observed (Table 2), which together led to an even higher increase in *E*_*Hertz*_, consistent with prior work^42^ (see the Supplementary Information Section E for details).

**Table 2:** Viscoelastic parameters of MDA-MB-231 cells without (control) and with doxycycline induction (Syk-expressing).

MDA-MB-231 cell line	*E*_*Hertz*_ (kPa)	*E*_0_ (kPa)	*α*
Control (N = 69)	0.55 ± 0.39 (0.44 ± 0.17)	1.3 ± 0.7 (1.3 ± 0.4)	0.17 ± 0.06 (0.16 ± 0.04)
Induced with doxycycline (Syk-expressing) (N = 69)	0.74 ± 0.40 (0.63 ± 0.21)	1.6 ± 0.7 (1.5 ± 0.4)	0.15 ± 0.04 (0.14 ± 0.03)

N is the number of measured cells. Mean ± s.d. (median ± m.a.d.). All cells were at 60–80% confluence.

In the second case study, we investigated alterations in the mechanical properties of normal mouse mammary epithelial cells (NMuMG) during epithelial-to-mesenchymal transition (EMT). NMuMG cells are extremely sensitive to TGF-β (known driver of EMT), undergoing morphological changes upon TGF-β treatment. Previous research with an AFM microrheology technique showed that *α* values decrease after TGF-β-induced EMT^43^. The same trend was observed here (Table 3). Moreover, the *E*_*0*_ value was approximately preserved during the transition, while *α* values decreased almost two-fold (see the Supplementary Information Section E for details). Such solidification of cells is probably associated with the assembly of stress fibers and increased pre-stress stored in the membrane and actin cortex^43^.

**Table 3:** Viscoelastic parameters of NMuMG cells before and during EMT.

NMuMG cell line	*E*_*Hertz*_ (kPa)	*E*_0_ (kPa)	*α*
Monolayer (epithelial state) (N = 62)	1.9 ± 1.4 (1.3 ± 0.6)	5.4 ± 3.1 (4.8 ± 2.2)	0.18 ± 0.06 (0.18 ± 0.04)
48 h TGF-β1 induction (N = 67)	4.0 ± 3.4 (3.0 ± 2.1)	8.0 ± 5.1 (6.7 ± 3.0)	0.15 ± 0.08 (0.12 ± 0.03)
96 h TGF-β1 induction (N = 68)	3.8 ± 2.6 (3.2 ± 1.2)	6.4 ± 4.4 (5.1 ± 1.8)	0.10 ± 0.02 (0.10 ± 0.01)

N is the number of measured cells. Mean ± s.d. (median ± m.a.d.).

## Discussion

Viscoelastic properties of cells have been investigated by AFM using several different experimental setups. In the time domain, creep and stress relaxation experiments have been conducted in a large number of studies^23, 44, 45^. Generally, a Heaviside step-loading is assumed before the hold phase (step-hold experiments)^46–48^ which greatly simplifies the analysis. However, in an actual experiment, the maximum loading speed is limited by hydrodynamic and inertial effects and step-loading conditions are impossible to implement. The fit of the total ramp-hold curve^49^ or introduction of the ramp correction factor should be implemented for proper analysis^50, 51^. Difficulties in interpretation may also arise at long relaxation times due to thermal drift in the instrument^52^ and from active cell responses to the applied forces^53^.

AFM experiments in the frequency domain are also popular for measuring cell viscoelastic properties. Here the cantilever is sinusoidally oscillated with fixed small amplitude and several frequencies during the period of indentation^2, 25–27, 43, 54, 55^, or during the scanning process^28, 30^. Frequency-dependent complex Young’s or shear modulus is measured from the amplitude and phase shift of the cantilever displacement. Such experiments generally require more experienced users and have their own set of uncertainties. Thus the extraction of cell viscoelastic properties directly from force curves remains by far the most easily implemented and attractive option for users. Several authors attempted to assess viscoelastic properties directly from force curves, by analysis of approach-retraction hysteresis^11, 20, 35, 56, 57^ or indentation-rate dependency of apparent elastic modulus^11, 36, 58^. However, the obtained parameters are not based on a complete viscoelastic solution like the Ting’s model and are thus defined as “apparent”. The main advantage of the method described here is that it has a firm theoretical foundation for viscoelastic analysis – Ting’s model – and can be applied directly to conventional AFM force curves. Therefore, it shares the limitations and uncertainties of the usual AFM experiments, including cantilever stiffness and optical lever sensitivity calibration^5^. Other limitations are absence of strong adhesion forces during the retraction; drifts and active cell response at low indentation speeds; relatively high computational costs. The method could be applied to the most indentation experiments (ramp-hold, sine function with a single peak), where indentation and force histories are available, conducted not only with AFM. All indentation data points are used for processing and no lag times before measurements are required. Measurement time may be adjusted by varying the piezo speed; fast force curves might be analysed with appropriate sampling rate. Different viscoelastic relaxation models can be used and compared. The method may further benefit from instruments with direct measurement of cantilever deflection and velocity as in laser Doppler vibrometry^59^.

This work clearly demonstrates that elastic contact mechanics is insufficient to describe force curves on live cells. What, if any, are the drawbacks to using *E*_*Hertz*_ to distinguish cell lines and phenotypes? Because of the ongoing relaxation, the Hertz’s model provides some effective, or apparent, value of Young’s modulus, *E*_0_ < *E*_*Hertz*_ < *E*_∞_, which depends on the indentation rate. Since *E*_*Hertz*_ is determined by the combination of *E*_0_ and *α* values, different cells with the same *E*_*Hertz*_ value can have different combinations of *E*_0_ and *α* values. Another example, in the case of cancer cells, low *E*_*Hertz*_ values might be caused by both low *E*_0_ and high *α* values. Thus, *E*_*Hertz*_ cannot provide a unique characterization of the viscoelastic phenotype of cells.

Here we used consistency between output results as a criterion for the applicability of a relaxation model: the model describes data well if it yields the same viscoelastic parameters (*τ* or *α*) for different experimental conditions (different piezo speeds). The SLS model provided consistent results for PAAm hydrogels for indentation times > 0.1 s (indentation rates < 4 μm/s), but failed at shorter times, where other relaxation mechanisms are probably manifest (Fig. 3g). The PLR model described data for cells better than the SLS model for indentation times from 50 ms–6 s, corresponding to piezo extension rates of 64–8000 nm/s, which cover most of the general AFM indentation experiments^1, 34, 57^. However, this finding might not hold outside of this range of experimental conditions. Here, an increase in power law exponent was observed at the lowest used indentation time of ~25 ms. Indeed, it was shown that the PLR model with a single exponent does not cover complete time/frequency ranges in rheological experiments on cells, where two or three regimes with different power law exponents were observed^45, 60–62^. In this study, at the highest used piezo speeds, we likely see a transition to the regime with the higher power law exponent (0.75), associated with lateral bending fluctuations of semiflexible cytoskeleton filaments found both in cells and in F-actin solutions^62–64^.

The basis of the PLR model is the so called soft glassy rheology (SGR) theory, developed for soft glassy materials^65^. This theory assumes that the observed general scale-free behaviour is a natural consequence of disorder and metastability of material internal structures. The cytoskeleton may represents such a structure in cells^66^. It consists of many disordered elements, which are held together by weak attractive forces and, as a result, trapped in energy wells. Scale-free (power-law) rheology arises from a wide distribution of energy well depths and element lifetimes. The power-law exponent *α* is related to the effective temperature of the material (amount of the agitation energy in the system), which determines the probability of elements to jump between the energy wells and reflects the system’s dynamics. The jumps of elements between wells are the origin of fluid-like behaviour, and the higher effective temperature (power law exponent) leads to more pronounced fluid-like features of the material. Thus, in the limit of *α* = 0 soft glassy materials behave like elastic solids and in the limit of *α* = 1 behave like viscous liquids. The measured exponent value of live cells here and before^66^ suggests that their rheology resembles that of a soft glassy material close to the glass transition. SGR theory implies that changes in the level of internal disorder and the effective temperature associated with cytoskeleton contraction or remodelling modulate cell rheological behaviour, and thus can provide a conceptual framework for such processes like cell migration, wound healing, invasion, metastasis and embryonic development^66^. Past studies also found relationships between PLR parameters in different cell groups^2, 54, 66, 67^. The stiffest group displayed the lowest power-law exponent, whereas the softest group displayed the highest. Here we also can observe such correlations between modulus *E*_0_ and *α* for groups of benign and cancerous cells, however, not inside the single cell line population. Differences in cytoskeleton structure between benign and cancerous cells are well known, but the relationships between cytoskeleton structure and *E*_0_ and *α* parameters requires additional research.

Other relaxation models also could be used with the present method in a straightforward manner using corresponding *E*(*t*) functions. Generalized Maxwell model with two or more relaxation times increased the quality of the fit relative to the single relaxation time like in SLS model^44, 47, 50^. But it is not completely clear if it is really capturing some physical phenomena or quality of the fit increased just due to the introduction of additional fitting parameters. Poroelasticity based viscoelastic models could also be used with an approximate solution obtained by finite-element simulations^48, 68, 69^. However, poroelastic effects seem to be relatively small at the shallow indentation and timescales used here. Fractional variation of the SLS model was recently used for description of cell rheology^70^. Mathematical analysis shows that it produce relaxation spectra similar to that of the PLR model^71^, and the choice of the model is more a question of convenience and simplification of the analysis.

It should be noted that adhesion forces are not accounted for in the presented Ting’s model. However, in experiments conducted here adhesion forces were low (the ratio of the maximum adhesive force to the maximum loading force was below 5%) and presumably could be safely ignored. Larger adhesive forces, however, may be responsible for the prominent part of the approach-retraction hysteresis and more complicated models should be used for the description of *F*-*Z* curves^72–76^. Yet, sometimes it is possible to diminish the adhesive force between the probe and the cell surface by proper cleaning and hydrophobic modification of the probe^38^.

In summary, we presented here general method bringing rigorous viscoelastic analysis within the reach of conventional AFM force curves. The method is highly robust, does not require changes in experimental design and is compatible with findings from conventional AFM microrheology. We believe it greatly enhances the potential of wider use and adoption of AFM for cell mechanics assays.

## Material and Methods

### Cells

NIH 3T3, MDA-MB-231, and MCF-7 cell lines were cultured in Dulbecco’s modified Eagle’s medium (DMEM) containing 10% FBS and 1% antibiotic/antimycotic solution (Invitrogen, Carlsbad, CA) in a humidified 5% CO_2_ atmosphere at 37 °C. For the NMuMG cell line, DMEM containing 10% FBS, 2 mM L-glutamine, 100 U/ml penicillin–streptomycin, and 10 μg/ml insulin (Sigma-Aldrich, USA) was used. Experiments were also conducted with the line of MDA-MB-231 cells expressing Syk-EGFP upon incubation with tetracycline analog^77^; and the expression of Syk-EGFP was induced where indicated by the addition of 1 μg/mL doxycycline for 24 h. Epithelial-to-mesenchymal transition (EMT) was induced in NMuMG cells by addition of TGF-β1 (R&D Systems, USA) in a final concentration of 10 ng/mL for 48 and 96 h. Prior to AFM experiments, cells were plated on glass-bottom cell culture dishes (FluoroDish, WPI, FL) and grown for a period of 1–2 days to a final confluence of ~70%. NMuMG cells were also prepared at higher confluence (monolayer) for EMT experiments.

### Gel preparation

Solutions of acrylamide and bis-acrylamide in PBS were polymerized with 10% ammonium persulfate and TEMED (Sigma-Aldrich, USA). Concentrations of acrylamide (10%) and bis-acrylamide (0.01%, w/v) were chosen to prepare a PAAm gel with approximately 1–2 kPa Young’s modulus, which is close to the eukaryotic cell stiffness. For AFM experiments, a total solution volume of 75 μL was allowed to polymerize between circular 18 mm (top) and square 24 mm (bottom) glass coverslips (VWR, USA). The bottom coverslip was aminosilanized with 3-aminopropyltriethoxysilane (APTES, Sigma-Aldrich, USA) and further coated with 0.5 (v/v)% glutaraldehyde solution in PBS (Fischer Scientific, USA) for at least 30 minutes. The upper coverslip was silanized with dichlorodimethylsilane (DCDMS, Sigma-Aldrich, USA). After 30 minutes of polymerization, the top coverslip was removed and the sample was extensively washed with PBS. Prepared PAAm gels had a ~200 μm thickness. The PAAm gels were kept in PBS for several days before the experiments to reach equilibrium. AFM measurements were performed in PBS containing 0.1% Triton X-100 detergent (Sigma-Aldrich, USA) to decrease probe-gel adhesion.

### AFM

AFM measurements were performed using a commercial MFP-3D-Bio atomic force microscope (Asylum Research, Oxford Instruments, USA) mounted on an IX-71 inverted optical microscope (Olympus, Japan). The AFM is equipped with a heated stage and in experiments with cells the sample temperature was kept constant at 37 °C. Tipless AFM cantilevers CSC38 (rectangular, Micromash Inc., Estonia) or BL-TR400PB (triangular, Olympus/Asylum Research, USA) were modified with 5 μm diameter silicon dioxide beads (Microspheres-Nanospheres, Corpuscular, NY, USA). The bead was glued to the end of the cantilever using UV-curable glue (Optical Adhesive No. 71, Norland Products, USA) under control of the inverted optical microscope. The typical spring constant of both cantilevers is 0.02–0.05 N/m. The accurate value was determined using the laser Doppler vibrometry system (Polytec MSA-400 Micro System Analyzer from Polytec GmbH, Waldbronn, Germany) using the equipartition theorem as described in refs 78 and 79. The radius of the probe was calculated after scanning the test grating TGT01 (Micromash Inc., Estonia). Before and after measurements, the relationship between the photodiode signal and cantilever deflection (sensitivity factor S) was calibrated by recording several force curves at a bare region of the glass coverslip and averaging its slope. Typically, *F*-*Z* curves were taken at 2 μm/s piezo displacement speed along the Z axis using the closed loop feedback circuit, so the piezo movement was corrected with the capacitive sensors implemented into the scanner of the AFM. Data sampling rate was 2 kHz in most experiments, but higher for higher piezo speeds. The piezo displacement range was 2–5 μm (typically 3 μm) to obtain a non-contact region that is long enough for the baseline determination. The force set point was chosen individually for all samples to obtain maximal indentation depth around 500 nm. At least 60–70 cells per cell line (3 *F*-*Z* curves per cell above its central part, where the cell height is large enough to diminish substrate effects), and 3 PAAm gel samples (10 *F*-*Z* curves at 3 random locations for each sample) were analysed. In experiments with varied scanner displacement speeds the force curves were acquired in a random order, 3 curves per each speed.

The protocols for stress relaxation and microrheology experiments are described in the Supplementary Information Section C.

### Adaptation of Ting’s solution for the AFM force curve processing

Raw AFM *F*-*Z* curves are presented as raw photodiode signal (Δ*V*) measured in Volts versus *Z* scanner displacement in nm (displacement of the cantilever base). First steps in the *F*-*Z* curves preprocessing are generally the same and independent of the mechanical model used, including conversion of the Δ*V* signal to force units (nN), determination of the zero force level and the contact point position. The force acting on the cantilever is calculated by Hooke’s law as *F* = *k* * *q*, where *k* is a cantilever spring constant and *q* is the cantilever deflection. The latter one is determined from the raw photodiode signal (Δ*V*) with the sensitivity factor S [nm/V]: *d* = *S* * Δ*V*.

The zero force level (baseline) was determined from the precontact region of the approach curve with the linear fit, then subtracted from the whole curve. The contact point was determined by the bi-domain fitting procedure^36^. Part of the approach curve, including both the precontact and contact regions, was fitted with a two-regime regression model (linear for noncontact and Hertz model for contact region), both Young’s modulus in Hertz’s theory framework (*E*_*Hertz*_) and the location of the contact point (*Z*_0_) were simultaneously estimated, and indentation depth is calculated as *δ* = Z − Z_0_ − *d*. After that step, the resulted curves represent force vs indentation dependencies (*F-δ* curves).

Here, we also included correction for the effect of hydrodynamic drag forces for the *F*-*Z* curves obtained at piezo speeds higher than 2 μm/s. This effect manifested as the separation between precontact regions of approach and retraction curves, which increases with the piezo speed. We adapted the procedure from^80^ to account for the hydrodynamic forces. As shown in previous work^80, 81^, the hydrodynamics forces are proportional to the probe velocity and the probe-sample separation. First, the baseline was determined as a median between precontact regions of approach and retraction curves, then these regions were independently fitted with the polynomial function (second order), normalized per the probe velocity, and then calculated hydrodynamic force were subtracted from both approach and retraction curves (Fig. S1). Otherwise, the hydrodynamic drag will make some contribution into the approach-retraction hysteresis of the contact part.

The Hertz’s model, which is traditionally used for description of the indentation with a spherical indenter, has the following form:

5 $$F(\delta )=\frac{4\sqrt{R}}{3(1-{\nu }^{2})}{E}_{Hertz}{\delta }^{\frac{3}{2}},$$

where *R* is the effective radius of curvature of the probe-sample system, 1/*R* = 1/*R*_*probe*_ + 1/*R*_*sample*_, or, in an assumption of the flat sample surface, it is just a radius of the spherical probe. *E*_*Hertz*_ is the Young’s modulus and *ν* is the Poisson’s ratio of the sample (the probe is assumed to be infinitely rigid in comparison to the soft samples like cells). Models for other probe geometries were introduced later^33, 37^ and can be used in corresponding experimental setup (conical, pyramidal, cylindrical indenter and others).

Lee and Radok utilized the elastic–viscoelastic correspondence principle to obtain the solution for indentation of a viscoelastic sample when contact area increases with time^82^. According to this principle, if the solution of the elastic problem is known, then the solution to the corresponding viscoelastic problem may be found by replacing elastic modulus after introduction of appropriate hereditary integral operator:

6 $$F(t,\,\delta (t))=\,\frac{4\sqrt{R}}{3(1-{\nu }^{2})}{\int }_{0}^{t}\psi (t-\xi )\frac{\partial {\delta }^{\frac{3}{2}}}{\partial \xi }d\xi ,$$

where *ψ*(*t*) is a sample relaxation function and *ξ* is the dummy time variable required for the integration. Henceforth, we will use Young’s modulus relaxation function, or, in other words, time-dependent Young’s relaxation modulus, *E*(*t*)^83^ to preserve consistency with the initial Hertz’s model equation. Young’s (*E*) and shear modulus (*G*) are related through *G* = *E*/[2(1 + *v*)] and, since for cells Poisson’s ratio *v* = 0.5 is widely accepted, *G*(*t*) = *E*(*t*)/3. Accordingly, a different prefactor should be used in the Eq. (6) for the shear relaxation modulus. As mentioned earlier, the Lee and Radok solution holds true only for the case when contact area increases with time, for the approach and holding phases in the AFM indentation experiments. But as we showed here, usage of only the approach phase data for extraction of the viscoelastic parameters might lead to erroneous results (see the Supplementary Information Section F for details).

Ting presented a more general approach to solving viscoelastic contact problem^32^. This approach can be applied to an arbitrary history of the contact area. For the general approach-retraction indentation cycle with a spherical indenter, Ting’s solution of the considered viscoelastic problem has the form presented in equation 1. The first part of the equation is valid for the approach part of *F-δ* curve (0 ≤ *t* ≤ *t*_*m*_, *t*_*m*_ is a time then approach phase ends; contact area *a(t)* increases during this phase) and it is equal to the solution of Lee and Radok. The second part of the equation is valid for the retraction part (*t* > *t*_*m*_). Here, the auxiliary function *t*_1_ is introduced and defined by *a*(*t*_1_) = *a*(*t*), *t*_1_(*t*) < *t*_*m*_, meaning that the contact area at time *t* during retraction is equal to the contact area at time *t*_1_ during approach. *t*_1_ values should be found that satisfy the formula in the equation (2). Using the *t*_*1*_(*t*) function, the contact area *a*(*t*_*1*_(*t*)) and the effective indentation *δ*_*1*_(*t*_*1*_(*t*)) during retraction were calculated. The effective indentation could be imagined as the indentation relative to the relaxing sample surface, as opposite to the indentation *δ* relative to the initial sample surface before relaxation. The modelled and experimental *F-δ* curves (Figs 2c and 4) indicate that the force during retraction becomes zero before indentation *δ* returns to the zero value, but zero force means zero contact area and, therefore, zero effective indentation (see also Supplementary Movie 1). This occurs when sample relaxation rate is slower than the cantilever retraction speed, also leading to the approach-retraction hysteresis.

The equations (1) and (2) provide a relation between the force, *F*(*t*), the indentation, *δ*(*t*), which are both known experimentally, and the relaxation function *E*(*t*). The rate of the indentation raised to the power 3/2 $\frac{\partial {\delta }^{\frac{3}{2}}}{\partial \xi }$ was calculated by numerical differentiation of the ${\delta }^{\frac{3}{2}}(t)$ data and then smoothed with the moving average filter (5 points). It should be noted, that during general AFM indentation experiments, the vertical piezo (cantilever base displacement) speed is constant, so both loading and indentation speed vary nonlinearly. The force changes nonlinearly with time because of the nonlinear change of the contact area, and the indentation speed during indentation is less than the piezo speed because of the increasing cantilever deflection (also nonlinear) during the indentation.

In principle, it is possible to extract *E*(*t*) function by replacement of the integral with the Riemann sum and then by calculation of *E*(*t*) for each time point. However, due to the fact that the time derivative of the indentation data is noisy and not quite accurate in the proximity of contact point, the produced error is difficult to evaluate. Furthermore, even after acquisition of a result for *E*(*t*), it should be compared with known viscoelastic models. An alternative model-based method we propose is to directly begin with the chosen viscoelastic model for *E*(*t*) and to see if this model can represent well the experimental force curve after implementation of the fitting procedure. Here, we compared two relaxation models, which are quite simple but yet frequently used to describe viscoelasticity of cells, namely, standard linear solid (SLS, also known as the three-element model, the Zener model and Prony series with one element) and power-law rheology (PLR) models, described by the equations (3) and (4) respectively. For the SLS model *E*_0_ is the instantaneous modulus [*E*_0_ = *E*(*t* → 0)], *E*_∞_ is the infinite (long-term, equilibrium) modulus [*E*_∞_ = *E*(*t* → ∞)] and *τ* is the relaxation time of the material. For the PLR model one can find different representations in the literature from as simple as *E*(*t*) = *E*_1_ *t* ^−α^ (*E*_1_ is modulus at *t* = 1 s)^39^. Here we used the following expression for the modified power law^71, 84^, which removes zero time singularity and allows more direct comparison with the SLS model:

7 $$E(t)={E}_{\infty }+\frac{{E}_{0}-{E}_{\infty }}{{(1+\frac{t}{t^{\prime} })}^{\alpha }},$$

where *E*_0_ and *E*_∞_ have the same meaning as in SLS model, *t*′ is a small time offset (here was set equal to the sampling time 5*10^−4^ s), and *α* is the power law exponent. *E*_∞_ is often considered to be 0 for cells especially in the rheology experiments, here we also adopted this assumption. We have also tried to leave it as a fitting parameter, and values close to 0 were obtained in most cases (data not shown). So the final expression looks simpler and only two parameters *E*_0_ and *α* determine the relaxation behaviour (Equation 4). Since *t*′ parameter is small relative to the time of the experiment, this equation is almost equal to the initial power law equation *E*(*t*) = *E*_1_ *t* ^*−α*^, and *E*_1_ could be calculated as *E*_1_ ≈ *E*_0_ *t*′^α^.

To extract the viscoelastic parameters from the experimental data, we used a fitting procedure with adaptations as described below. In the fitting procedure, the least squares error between the AFM indentation data and the models described above is minimized,

8 $$e=\sum _{i=1}^{n}{({F}_{i}^{expt}-{F}_{i}^{Ting})}^{2},$$

where *e* is an error function (norm of residuals), which together with coefficient of determination R^2^ provides the information about goodness of fit with chosen relaxation model. The benefit of such method is that all data points in curve are used, and if some of them are affected by noise, the contribution of the noise will be diminished proportionally to the total number of data points. The non-linear Levenberg–Marquardt or the trust-region-reflective least squares algorithms is often used for such nonlinear optimization problem. The latter one showed better results in our preliminary convergence analysis and therefore was used in this study. The algorithm was implemented in the MATLAB (MathWorks, USA), and all integrals were evaluated numerically in the same software. Implementation of numerical analysis might require high sampling rates (small time steps Δ*t*), but here we did not noticed effects of sampling rates on obtained results in the range 100–4,000 Hz (Δ*t* = 0,01–0,00025 s) for 2 μm/s piezo velocity, so 2 kHz sampling frequency was used. It gives approximately 1 point per 1 nm of piezo displacement, the same ratio was preserved for the higher piezo velocities.

We noticed that the best fit was obtained by adjusting the contact point position slightly. The initial estimate for the contact point was obtained with the elastic assumptions of the Hertz’s model and might not be equal to the optimal contact point position for the viscoelastic model. Henceforth, the contact point position was varied in the region close to the initial estimate, then the solution providing the lowest norm of residuals was chosen as the final position^85^ (see the Supplementary Information Section A for details). Such behaviour is probably associated with the uncertainties in the beginning of the approach phase caused by long-range electrostatic forces, steric forces from the brush layer on the surface of cell^86^, surface roughness, or contamination of the probe surface – all factors that mostly affect the beginning of the approach curve, but whose contributions decrease with indentation depth. Indeed, the resulting fit shows that larger residuals are observed in the beginning of the curve (Fig. S3). In experiments with varied indentation depths (Figs S10 and S16), strong variation of viscoelastic parameters were also found at small depths.

The algorithm presented here could be combined with bottom-effect correction models for the sample of a finite thickness in a straightforward manner (see the Supplementary Information Section A for details). The custom MATLAB codes used in the analyses are available from the corresponding author upon request.

### Finite element analysis

Finite element (FE) simulations were conducted in Abaqus CAE (version 14, Simulia Corp., Providence, RI). The axisymmetric system consisted of the rigid spherical indenter with 2 μm radius and the viscoelastic cylindrical sample with 15 μm height and radius (Fig. 2). The indentation depth and speed were selected to be 500 nm and 1 μm/s respectively, both for approach and retraction. The sample was meshed more finely in the contact region. The bottom surface of the sample was constrained, and the contact between the indenter and the sample was considered to be frictionless.

*E*_0_ of the sample was set to 2 kPa. For SLS model *τ* was set to 0.1 s and *E*_∞_ to 1 kPa. PLR model could not be directly prescribed in this FE software, so it was approximated as the Prony series expansion including six terms, with coefficients adjusted in MATLAB. The effectiveness of such approach was showed previously^87, 88^. Power law exponent *α* was chosen to be 0.2. More details could be found in the Supplementary Information Section B.

### Statistical Analysis

All statistical analyses were performed using OriginPro 2016 software (OriginLab Corporation, MA). A 2-tailed Pearson’s correlation coefficient was used to characterize the correlation between measured viscoelastic parameters. A non-parametric Mann–Whitney U test was used to determine the statistically significant differences between the groups. Since most of the data were distributed not normally, results are presented both as mean ± standard deviation and median ± median absolute deviation. The percentiles in the box-and-whisker plots are 10%, 25%, 50%, 75% and 90%, the dots are the outliers.

## Electronic supplementary material

Supplementary Movie 1

Supplementary Information
