# Supplementary material for: Measuring nanoscale viscoelastic parameters of cells directly from AFM force-displacement curves
Source: Sci Rep. 2017 May 8;7:1541. doi: 10.1038/s41598-017-01784-3 (PMC5431511; doi:10.1038/s41598-017-01784-3)
Supplement: Supplementary file 2 — Supplementary Information [file 41598_2017_1784_MOESM2_ESM.doc]

**Supplementary Information**

**for**

**Measuring nanoscale viscoelastic parameters of cells directly from AFM**

**force-displacement curves**

Yu.M Efremov^1,2^, W. Wang^3^, S.D. Hardy^3^, R.L. Geahlen^3,4^ and A. Raman^1,2^

^1^School of Mechanical Engineering, Purdue University, West Lafayette, Indiana 47907, USA

^2^Birck Nanotechnology Center, Purdue University, West Lafayette, Indiana 47907, USA

^3^Department of Medicinal Chemistry and Molecular Pharmacology, Purdue University, West Lafayette, Indiana, 47907, USA

^4^Purdue University Center for Cancer Research, Purdue University, West Lafayette, Indiana, 47907, USA

. Correspondence and requests for materials should be addressed to A.R. (email: raman@purdue.edu)

The sections of this document are:

1. Application of the Ting’s model to experimental AFM force curves
2. Finite Element Simulation
3. Comparison with other methods
4. Additional data for the hydrogels
5. Additional data for the studied cell lines
6. Additional information for the developed method
7. **Application of the Ting’s model to experimental AFM force curves**

The following equations describe Ting’s solution^1^ (see also^2,3^) for indentation of a viscoelastic sample of a finite thickness *H* with a rigid indenter:

$F\left(t,\delta\left(t\right)\right)=\left\{\begin{matrix} \frac{2C_{n}}{\left(1-\nu^{2}\right)}\int_{0}^{t} E\left(t-\xi\right)\frac{\partial\delta^{\frac{n+1}{n}}}{\partial\xi}f_{n}\left(\delta\right)d\xi, 0\leq t\leq t_{m} \\ \frac{2C_{n}}{\left(1-\nu^{2}\right)}\int_{0}^{t_{1}\left(t\right)} E\left(t-\xi\right)\frac{\partial\delta^{\frac{n+1}{n}}}{\partial\xi}f_{n}\left(\delta\right)d\xi, t\geq t_{m}\geq t_{ind} \end{matrix}\right.$, (1)

$\int_{t_{1}\left(t\right)}^{t} E\left(t-\xi\right)\frac{\partial\delta}{\partial\xi}d\xi=0$, (2)

where *F* is the force acting on the cantilever; *δ* is the indentation depth; $t$ is the indentation time initiated at initial contact ($t_{m}$is the duration of approach phase, $t_{ind}$ is the duration of complete indentation cycle); *ξ* is the dummy time variable required for the integration; $E\left(t\right)$ is the Young’s relaxation modulus; $\nu$ is the Poisson’s ratio of the sample (assumed to be time-independent); $n$ and $C_{n}$ are constants related to indenter shape: $n=0$, $C_{0} = R_{с}$ for cylindrical punch ($R_{с}$ is the radius of cylinder); $n=1$, $C_{1} = \left(tan \alpha\right)/\pi$ for conical indenter (*α* is the included half-angle of cone; similarly, for pyramidal indenter $C_{1} = 1.4906*\left(tan \alpha\right)/4$, where *α* is the opening angle of the pyramid^4^), $n = 2$, $C_{2} = 2\sqrt{R}/3$ for spherical indenter ($R$ is the radius of sphere); and $f_{n}$ is the bottom-effect correction factor, which depends only on the indentation depth^5–7^. $f_{n}=1$ in the assumption of the infinite sample thickness. As shown in FEM simulations, the bottom-effect correction factor is independent from the loading history^5^, so it does not depend on the material relaxation model but only relates to the geometric parameters of indenter shape, indentation depth and sample height (thickness). In this way the model can be applied to force-volume maps, from which sample height in each point is known. Hereafter, we will imply the model for a spherical indenter, which was used in our experiments.

The equations (1) and (2) are sufficient to model the force-indentation curve for the prescribed indenter parameters, indentation function $\delta\left(t\right)$*,* the Young’s relaxation modulus $E\left(t\right)$, thickness and the Poisson’s ratio of the sample (Fig. 1 and S2). The task is, however, to extract the viscoelastic parameters from the experimental force-displacement (*F-Z*) curve.

As described in the manuscript and in the previous work^8^, during the preprocessing steps raw *F-Z* curves, which presented in units of photodiode signal (∆V) versus vertical scanner displacement ($\Delta Z$), were converted to the force versus indentation dependencies (*F-δ* curves). We also included a correction for the effect of hydrodynamic drag forces for the curves obtained at piezo speeds higher than 2 μm/s (Fig. S1). This effect is manifest as the separation between precontact regions of approach and retraction curves, which was larger for triangular than rectangular cantilevers and increased with the piezo speed. We adapted procedure from^9^ to account for the hydrodynamic forces. As shown in previous works^9,10^, the hydrodynamics forces are proportional to the probe velocity and the tip-sample separation. First, the baseline was determined as a median between precontact regions of approach and retraction curves, then these regions were independently fitted with the polynomial function (second order), normalized per the probe velocity, and then the calculated hydrodynamic forces were subtracted from both approach and retraction curves. Otherwise, the hydrodynamic drag will contribute to the approach-retraction hysteresis and will introduce error in calculated viscoelastic parameters. The corrected *F-Z* curves were next processed the same way as other raw curves.


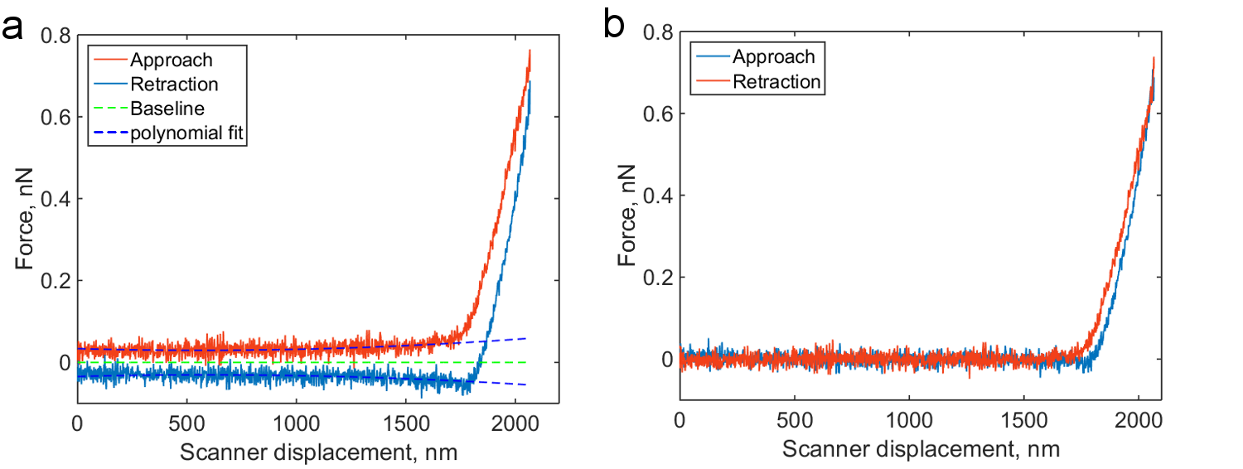


**Figure S1. Hydrodynamic drag effect correction for the *F-Z* curves, scanner displacement rate 8 μm/s. a,** Hydrodynamic drag effect is manifest as a separation between precontact parts of the *F-Z* curve. The baseline and the second order polynomial function fits for precontact regions are shown. **b,** Corrected force curve.

After this preprocessing step, we have force *F* versus indentation *δ* dependency and also $F^{expt}\left(t\right)$ and $\delta\left(t\right)$ time histories since AFM sampling rate is known (Fig. S2 and S3A-C), $t=0$ is assigned to the contact point. To model the curve with the Ting’s equations, for each data point of the *F-δ* curve functions $\frac{\partial\delta}{\partial\xi}(t)$ (indentation speed) and $\frac{\partial\delta^{\frac{3}{2}}}{\partial\xi}(t)$ were calculated by numerical differentiation of the $\delta\left(t\right)$ and $\delta^{3/2}\left(t\right)$ data respectively and then smoothed with the moving average filter (5 points) (Fig. S3D, E). Relaxation function $E\left(t\right)$ were constructed from the chosen relaxation model with some initial guesses (e.g. $E_{0}=2E_{Hertz}$, *α* = 0.1) for the viscoelastic parameters. For each data point of the approach curve, Ting’s model (Eq. 1) was used to calculate $F^{Ting}\left(t\right)$ values, integrals in Ting’s equations were calculated numerically with the trapezoidal rule. Next, the $t_{1}\left(t\right)$ function was numerically calculated for each data point of the retraction curve by finding $t$ value among the points of the approach curve that provides the closest to 0 value of the corresponding integral (Eq. 2). With $t_{1}\left(t\right)$ function, Ting’s equations for the retraction curve was calculated by numerical integration and complete $F^{Ting}\left(t\right)$ function was obtained.

The next step is to adjust the input viscoelastic parameters to obtain the modelled $F^{Ting}\left(t\right)$ function as close as possible to the experimental $F^{expt}\left(t\right)$ function; for that, the nonlinear optimization algorithm (the fitting procedure) was used. The algorithm minimizes the least squares error (norm of residuals) between the AFM indentation data and the model:

$e=\sum_{i=1}^{n} (F_{i}^{expt}-F_{i}^{Ting})^{2}.$ (3)

The non-linear Levenberg–Marquardt or the trust-region-reflective least squares algorithms are often used for such nonlinear optimization problem. The latter showed better results in our preliminary convergence analysis and therefore was used for this study. The output of the algorithm is the set of viscoelastic parameters of the chosen viscoelastic model that describe the force curve in the best way. All described numerical procedures were implemented in MATLAB (MathWorks, USA), examples with the results of the fit are shown in the manuscript (Fig. 4).


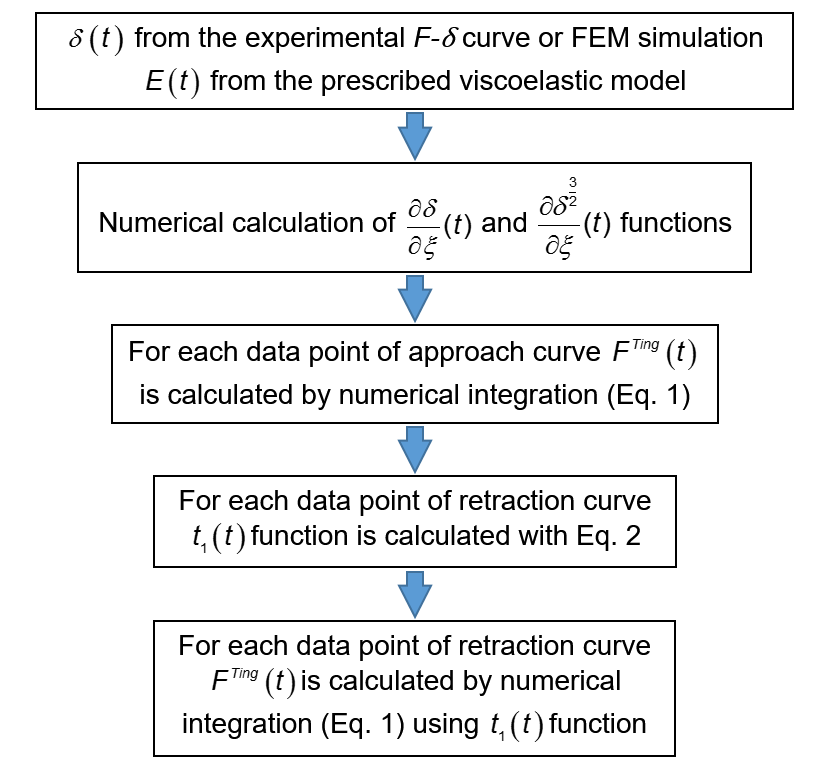


**Figure S2. Flowchart of the algorithm for numerical construction of the force-indentation curve with the Ting’s model.**

For the experimental force curves, we also found that the best fit could be obtained by adjusting the contact point position slightly. Initial position for the contact point was obtained with the elastic assumptions of the Hertz’s model and might not be equal to the optimal contact point position for the viscoelastic model. Henceforth, contact point position was varied in the region close to the initial position, then solution providing the best fit (the lowest error) was chosen as the final position (Fig. S4A). Systematic trend in the final contact point position shift toward deeper locations was found (10-80 nm). This could be a region affected by the relatively long-range interactions which are excluded based on the best fit. Indeed, the largest residuals were observed close to the contact point position in the beginning of the indentation (Fig. S4B).

**
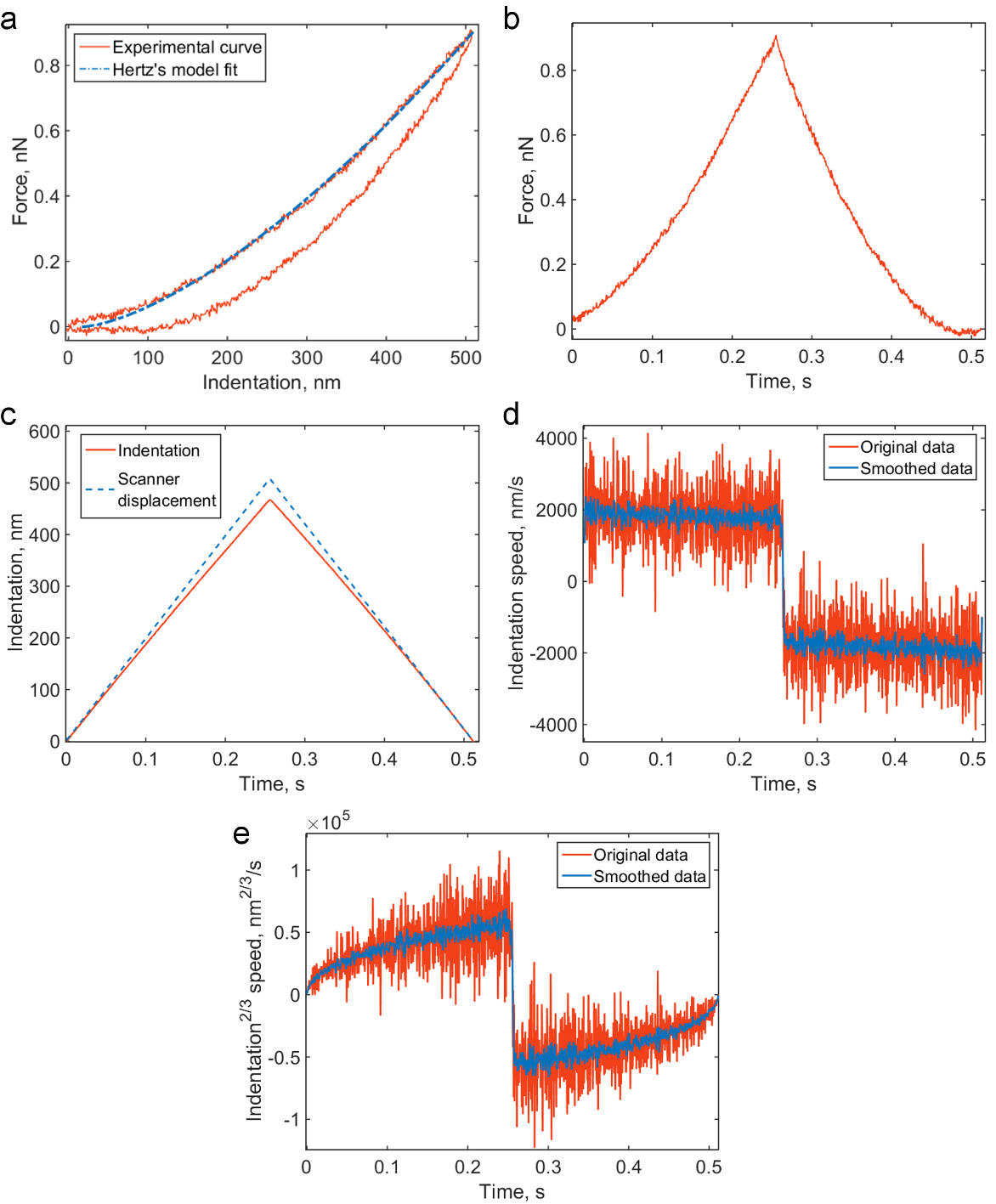
**

**Figure S3. Acquisition of input data for the algorithm. a,** Force-indentation (*F-δ*) curve obtained on NIH 3T3 fibroblast, Hertz’s model fit is shown. **b,** Force vs time curve. **c,** Indentation vs time curve, scanner displacement is shown for comparison. **d,** Calculated indentation speed. It is close to the used scanner displacement speed 2 μm/s but lower due to cantilever deflection. **e,** Speed of the indentation raised to the power 3/2, used in the Ting’s equations.


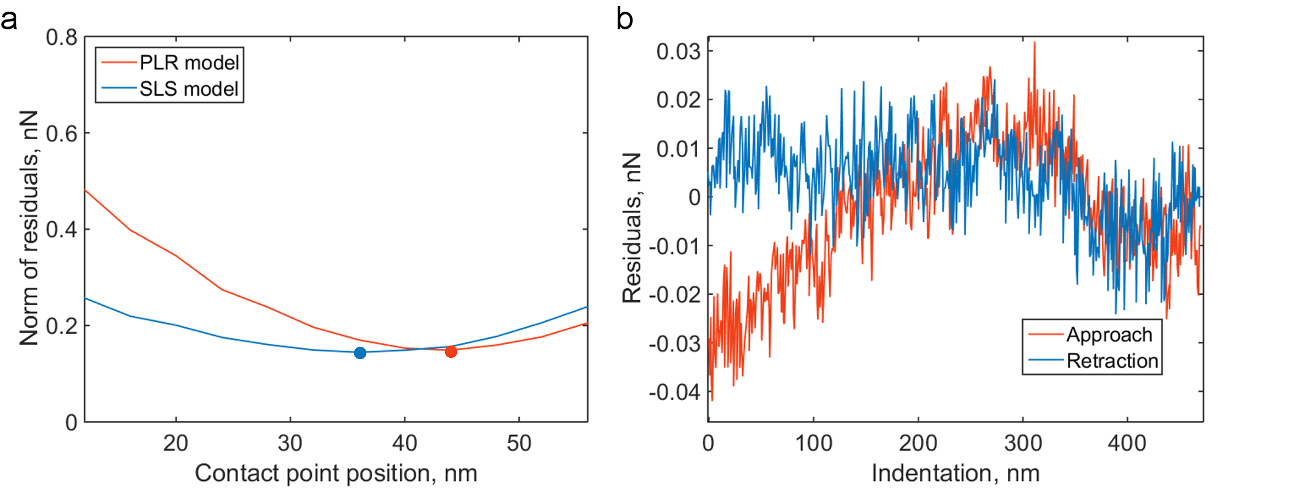


**Figure S4. Determination of the optimal contact point position. a,** The graph is showing fit error versus shift of the contact point relative to the initial position (Hertz’s model fit result) for PLR and SLS models for experimental force curves showed in Fig. S3. Optimal position providing the lowest fit error is marked (44 nm for PLR model and 36 nm for SLS model). **b,** The residuals of the best fit for the force curve showed in Fig. S3, PLR model. The largest residuals are in the area of beginning of the indentation.

We checked assumptions about linear viscoelastic behavior of cells and gels. If it is correct, the extracted viscoelastic parameters should not depend on the indentation depth within reasonable limits^10^. Indeed, variations in the indentation depth on the PAAm hydrogels and cells in the range of 300-1,000 nm did not lead to significant changes in measured parameters, except for some variations observed at low (<200 nm) indentations, probably caused by long-range forces and uncertainties in this region (Fig. S4B, Fig. S10, Fig. S16).

It should be noted that adhesion forces are not accounted for in the presented Ting’s model. However, in experiments conducted here, the ratio of the maximum adhesive force to the maximum loading force was below 5%. Based on criteria suggested by Johnson^11^, this means that adhesion does not affect the measurements significantly. Larger adhesive forces, however, may be responsible for the prominent part of the approach-retraction hysteresis and could not be safely ignored. For indentations on cells with a spherical probe, a JKR type contact is generally relevant, although even more complicated models are required, there adhesion by itself is indentation time- and rate-dependent^12^. Viscoelasticity models in conjunction with such adhesion models are significantly more complicated^13–16^, and application of such models for measurements on cells is the aim of future research. Yet, it is possible to diminish the adhesive force between the probe and the cell surface by proper cleaning and hydrophobic modification of the probe^17^.

1. **Finite element simulation**

We used finite element (FE) analysis to validate the derived algorithms. FE simulations were conducted in Abaqus CAE (version 14, Simulia Corp., Providence, RI). The axisymmetric system consisted of the rigid spherical indenter with 2 μm radius and the viscoelastic cylindrical sample with 15 μm height and radius. The bottom surface of the sample was constrained, and the contact between the indenter and the sample was considered to be frictionless. The indentation depth and speed were selected to be 500 nm and 1 μm/s respectively, both for approach and retraction. The sample was meshed with 4-node bilinear axisymmetric quadrilaterals CAX4R (contact region) and 3-node linear axisymmetric triangles CAX3, more finely in the contact region, the total number of elements was 48,335. The number of elements was adjusted to obtain reliable accuracy and simulation time in the preliminary analysis.

$E_{0}$ of the sample was set to 2 kPa for both models. For SLS model, *τ* was set to 0.1 s and $E_{\infty}$ to 1 kPa, the Poisson’s ratio was 0.48. PLR model could not be directly prescribed in this FE software, so it was approximated as the Prony series expansion including six terms, with coefficients adjusted in the MATLAB (MathWorks, USA) (Fig. S5). The effectiveness of such approach was showed previously^5,18^. Power law exponent *α* was chosen to be 0.2. For this FE simulation, geometry and distribution of effective von Mises stress during the indentation process could be found in Supplementary Movie 1.


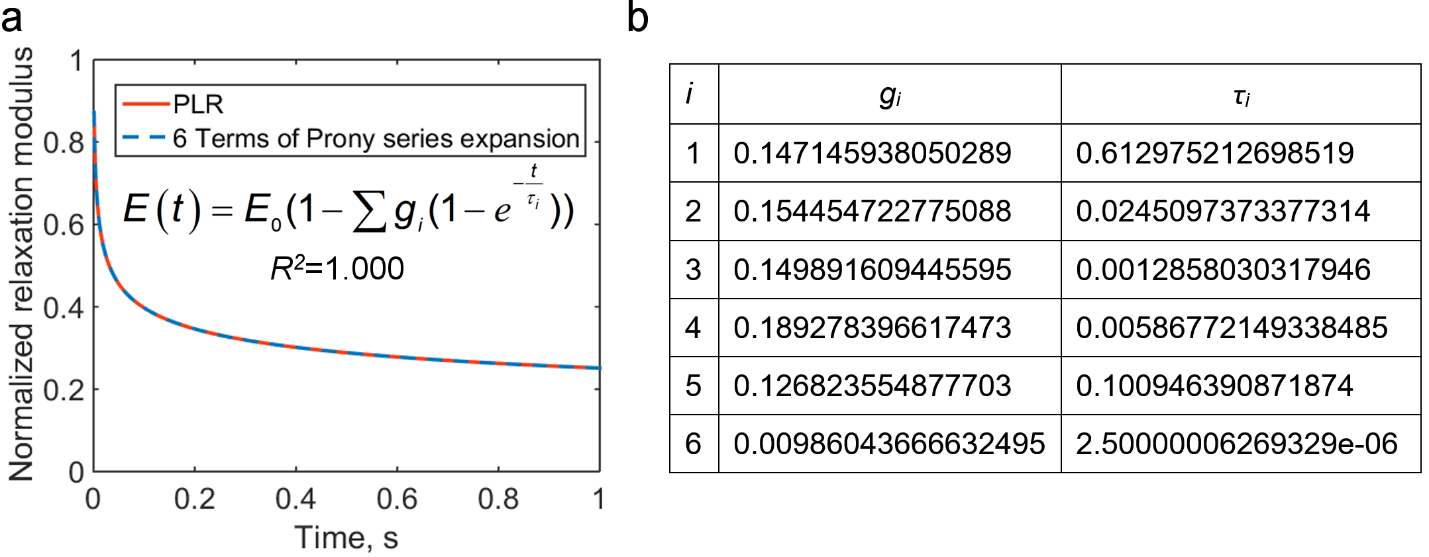


**Figure S5. Approximation of the PLR relaxation model with the Prony series expansion including six terms.** **a**, Normalized relaxation modulus ($E/E_{0}$) for PLR model and its fit with the six-term Prony series. **b**, Coefficients for the terms in the Prony series obtained from the fit and used in the FEM simulation.

**Table S1 Results of fitting of the FE simulation data.** FE model input parameters are: $E_{0}$ = 2,000 Pa, $E_{\infty}$ = 1,000 Pa, *τ* = 0.1 s for SLS model, $E_{0}$ = 2,000 Pa, *α* = 0.2 for PLR model. *Data in parentheses are acquired with the bottom-effect correction.

|  | **Sample with 15 μm thickness,**  **no spring** | | **Sample with 3 μm thickness,**  **no spring** | | **Sample with 15 μm thickness,**  **spring 0.01 nN/nm** | |
| --- | --- | --- | --- | --- | --- | --- |
|  | SLS | PLR | SLS | PLR | SLS | PLR |
| ***E_0_* (Pa)** | 2,058 | 2,040 | 2,610  (2,043)* | 2,934  (2147) | 2,020 | 2,039 |
| ***E_∞_* (Pa)** | 1,037 |  | 1,312  (1,026) |  | 1,034 |  |
| ***τ*(s) or *α*** | 0.10 | 0.198 | 0.1548  (0.118) | 0.204  (0.197) | 0.105 | 0.198 |

Model-predicted and FE-simulated curves were markedly close to each other (Fig. S6). Some discrepancy appeared at higher indentation depths and might be associated with finite-size effects and with the approximate nature of the Hertz’s model (paraboloid vs sphere, assumption $\delta<<R$). Moreover, an excellent agreement between FE parameters and the results of fitting procedure (R^2^ > 0.99) were observed for both SLS and PLR models, for all of them the difference between model and simulation was below 4% (Table S1).

In the real AFM experiments indentation speed changes during the indentation process due to the cantilever deflection. To simulate the AFM experiments more closely, the spring element was added that connected the probe and the additional base element. The movement was prescribed to the base with the same 1 μm/s speed and 500 nm displacement. The stiffness of the spring was set to 0.01 nN/nm. Again, model-predicted and FE-simulated curves (force vs scanner displacement, indentation vs time, indentation speed vs time, contact area vs time) were similar (Fig. S6) and the fitting provided close parameters (the difference < 4%, Table S1). The indentation speed decreased at higher depths and was affected most prominently in the beginning of retraction.

The sample thickness was decreased to 3 μm to check the effect of the finite sample size. Without correction, the values of $E_{0}$ (for both SLS and PLR models), $E_{\infty}$ and *τ* were overestimated by ≈50%. Interestingly, the lower thickness did not affect *α* value noticeably. After the introduction of the correction factor^6^, the fit provided correct parameters (≈3% difference) except *τ*, which still was ≈20% overestimated. Lower overestimation (4%) was obtained when indentation depth was reduced to 300 nm.


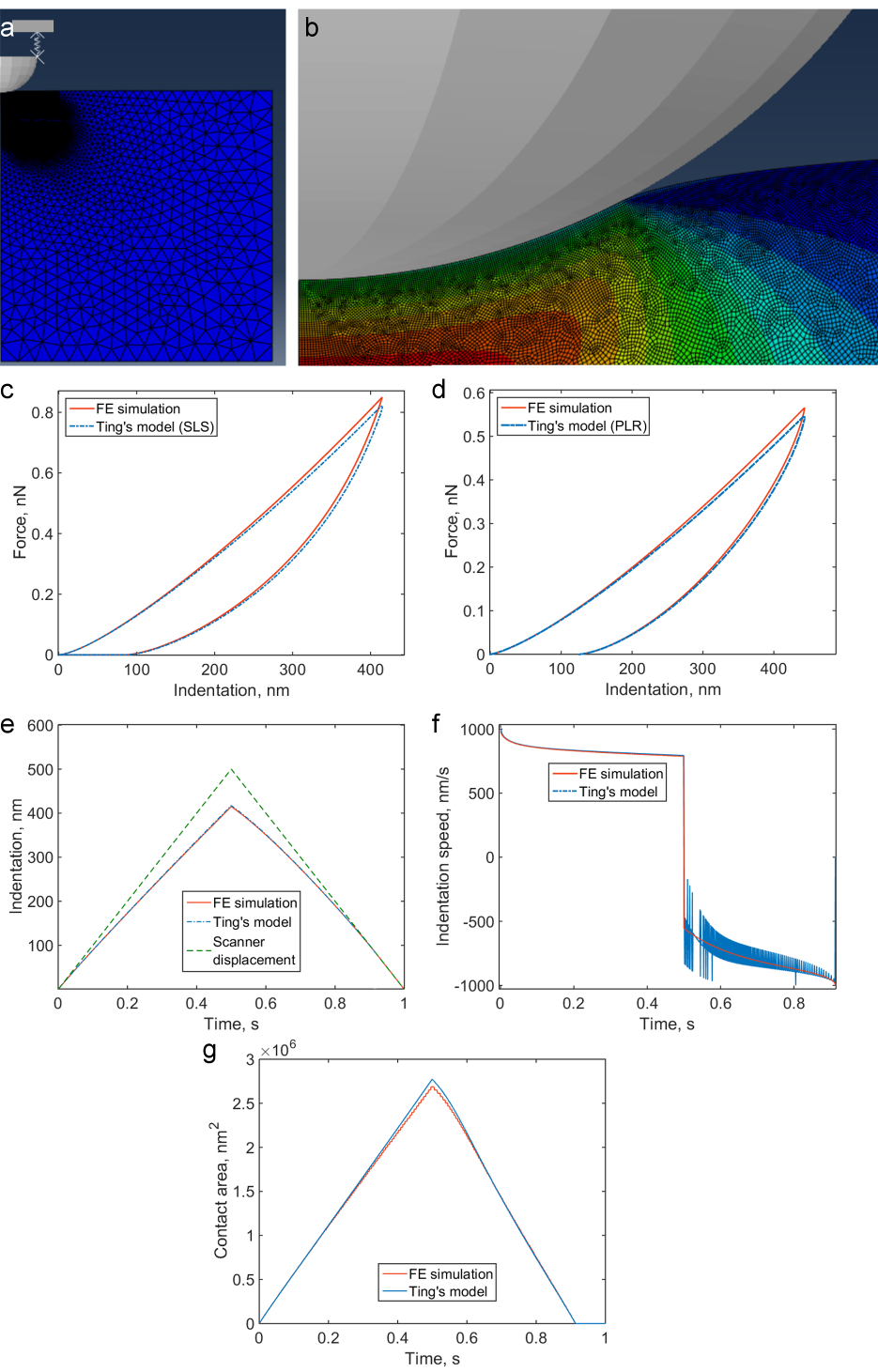


**Figure S6. Finite element (FE) simulation of AFM indentation experiments. a,** Axisymmetric FE model: the rigid spherical indenter with 2 μm radius connected to the moving base via spring and the viscoelastic cylindrical sample with 15 μm height and radius. **b,** Magnified view of stress distribution in the sample under the maximum load. **c,** *F-δ* curve (FE simulation and Ting’s model) for SLS model. **d,** *F-δ* curve for PLR model. All next presented curves are for SLS model. **e,** Indentation vs time curve, scanner displacement is shown for comparison. **f,** Indentation speed vs time curve. Prescribed scanner displacement speed was 1 μm/s; indentation speed is lower due to cantilever deflection. **g,** Contact area versus time.

1. **Comparison with other methods**

To compare the presented method with the previous techniques (step-hold stress relaxation and microrheology) the experiments were conducted on PAAm hydrogels (3 samples) and NIH 3T3 cells (40 different cells) at the same locations in the random order. The force curves for the processing with the presented method were obtained at 2 µm/s piezo speed.

Step-hold stress relaxation experiments where conducted by protocol that is similar to the previously described^19^. Briefly, a dwell period with the constant Z-extension is added between approach and retraction phases of the force curve obtained at the 10 µm/s piezo speed. Duration of the dwell period was set to 1 s for both gels and cells. The indentation process was approximated by using the Heaviside step function, so the force during the dwell period were fitted with the analytical solution of the Lee-Radok equation:

$F(t)=\frac{4\sqrt{R}\delta_{0}^{\frac{3}{2}}}{3(1-v^{2})}E(t);$ (4)

where $\delta_{0}$ is the indentation during the dwell phase, which was regarded as approximately constant in time (change in deflection of cantilever was <5% with respect to the ≈500 nm indentation).

Viscoelastic behavior of cells in the frequency domain was characterized by the protocol described in^20,21^ with little modifications. Briefly, low-amplitude (25 nm) sinusoidal perturbations with a 0.3-81 Hz frequency (6 discrete frequencies) where applied to scanner Z-extension during the dwell period of the force curve in a random order. A complex Young’s modulus $E^{*}(f)=E'(f)+iE''(f)$ was computed with the correction for the probe-sample contact geometry^20^ (the first term of the Taylor expansion of the Hertz model was used) and for the hydrodynamic viscous drag^10^. *E**(*f*) data were fitted with the SLS model, which in frequency domain has the following form^22^:

$\begin{matrix} E'\left(f\right)=E_{\infty}+\left(E_{0}-E_{\infty}\right)\frac{(2\pi f)^{2}\tau^{2}}{1+(2\pi f)^{2}\tau^{2}}, \\ E''\left(f\right)=\left(E_{0}-E_{\infty}\right)\frac{(2\pi f)\tau}{1+(2\pi f)^{2}\tau^{2}}; \end{matrix}$ (5)

or with the power-law structural damping model^22–24^:

$E^{*}(f)=\bar{E}_{1}(1+i\eta)\left(2\pi f\right)^{\alpha}+if\mu;$ (6)

where $\eta=tan\left(\alpha\pi/2\right)$ is the hysteresivity or structural damping coefficient of the model, $\bar{E}_{1}$ is the scale factor for storage and loss moduli (it is equal to $E'(f)$ at $f=1/2\pi$ Hz), and *µ* is the Newtonian viscous damping coefficient. $\bar{E}_{1}$ in the frequency domain (and $E_{1}$ in the time domain (value of $E(t)$ function at $t=1$ s) are related through the next equation^22^:

$\bar{E}_{1}=\frac{\pi}{2\Gamma(\alpha)\sin(\frac{\alpha\pi}{2})}E_{1},$ (7)

where $\Gamma$ represents the Euler function. The $E_{1}$ parameter of PLR model was chosen for comparison between the methods.

For both stress relaxation and microrheology experiments, SLS and PLR models provided better fit for hydrogels and cells respectively (Fig. S7). Thus, SLS model parameters were compared for hydrogels and PLR model parameters were compared for cells. The comparison shows a reasonably good agreement between all three methods (Table S2).

In experiments on hydrogels, lowest values of $E_{0}$ were obtained with the stress-relaxation testing. This could be explained by the weakness of the Heaviside step approximation. Actual duration of the step was ~0.05 s at the used 10 µm/s piezo speed, which is comparable with the relaxation time of the hydrogel. Therefore, the beginning of the relaxation process is excluded from the analysis, leading to the underestimation of $E_{0}$. In the microrheology experiments, only the fit in the 0.3-3 Hz region provided close SLS model parameters to the other two tests, while usage of a wider region (0.3-81 Hz) led to increase in $E_{0}$, $E_{\infty}$ and decrease in *τ* values (data not shown). The same trends were observed with the Ting’s model for the force curves obtained at high piezo speeds (Fig. 3, Fig. S11) and indicate inappropriateness of SLS model for hydrogel description at high frequencies.


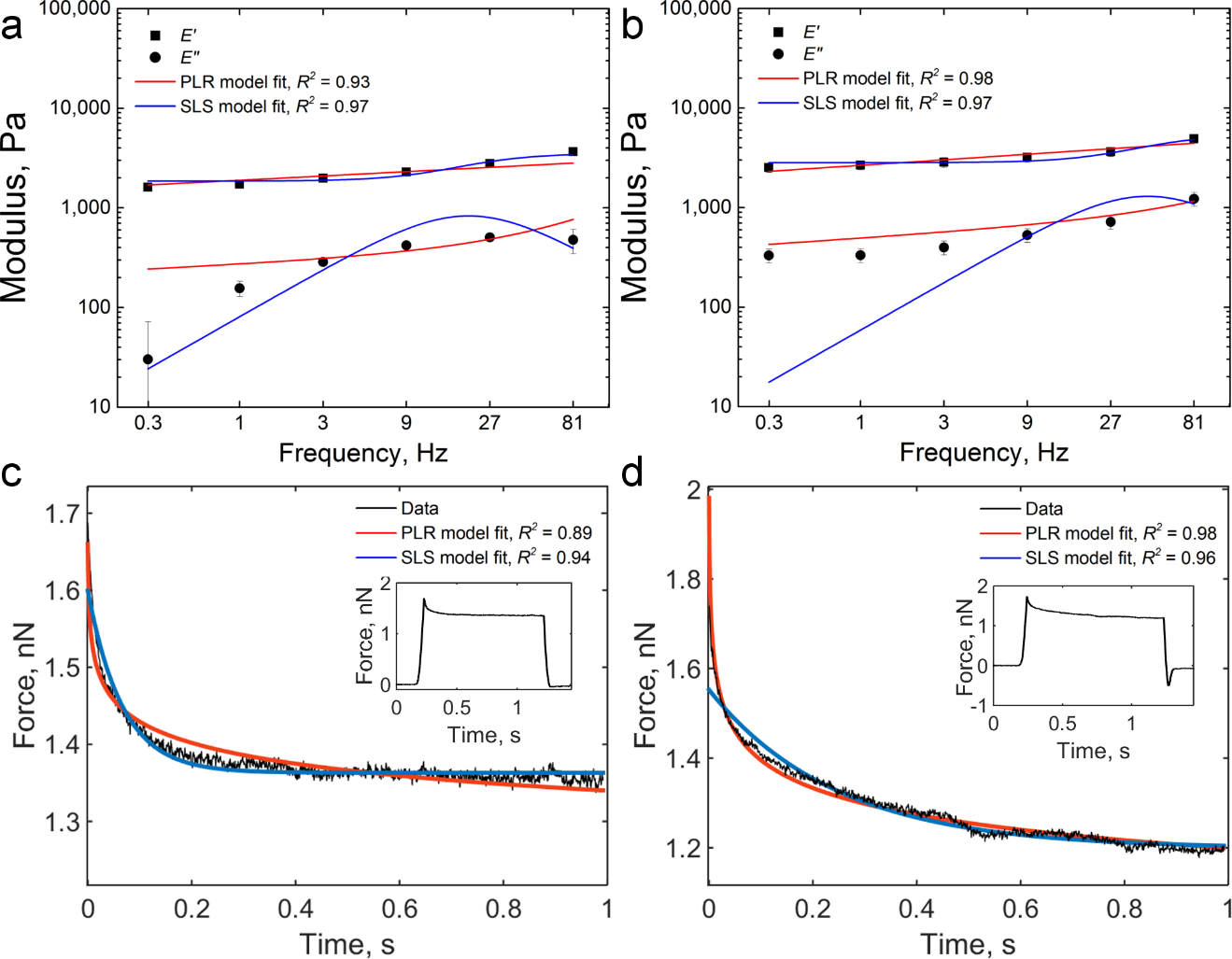


**Figure S7. Stress relaxation and microrheology experiments on PAAm hydrogels and NIH 3T3 cells*.* a, b** Frequency dependence of the storage $E'$ and loss $E''$ modulus measured on PAAm hydrogels (**a**, mean±SEM for 3 hydrogels) and NIH 3T3 cells (**b**, mean±SEM for 40 cells). PLR and SLS model fits are shown. **c,d** Representative examples of stress relaxation curves measured on PAAm hydrogels (**c**) and NIH 3T3 cells (**d**). PLR and SLS model fits are shown. Insets show corresponding full experimental force curve.

In experiments on NIH 3T3 cells, stress-relaxation testing provided lowest values of *α*, which again could be caused by the weakness of the Heaviside step approximation. In the microrheology experiments, width of the fit region affected PLR parameters only slightly, indicating appropriateness of the PLR model (data not shown). The value of Newtonian viscosity *µ* was 8±5 Pa s. For measured population of cells (40 cells) and used methods, strong correlation between PLR parameters were found (Pearson’s $r>0.71$, $p<10^{-7}$) (Fig. S8).


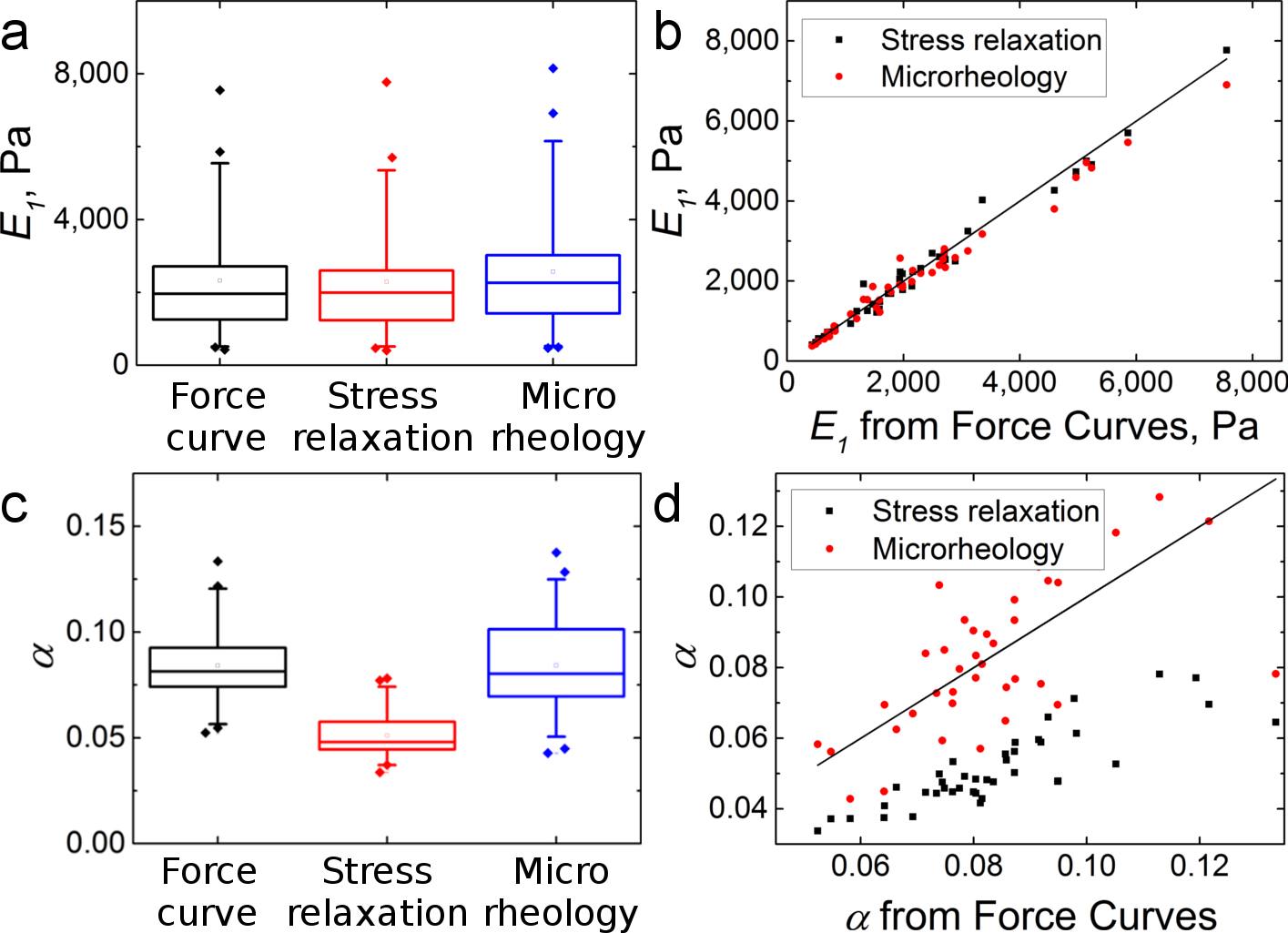


**Figure S8. Comparison of viscoelastic parameters from the 3 measurement methods on NIH 3T3 cells (40 cells total)*.* a,** Box plots of $E_{1}$ values. **b** $E_{1}$ values obtained from stress-relaxation and microrheology experiments versus $E_{1}$ values obtained from force curves **c,d** Same graphs for *α* values. Black line on **c** and **d** represent the $r=1$ correlation.

**Table S2. Comparison of viscoelastic parameters from the 3 measurement methods.** Data are mean±SD. *Parameters for microrheology experiments on gels obtained with the SLS model fit in the 0.3-3 Hz region, for NIH 3T3 cells in the 0.3-21 Hz region.

| Method | PAAm hydrogels,  SLS model | | | NIH 3T3 cells,  PLR model | |
| --- | --- | --- | --- | --- | --- |
|  | $E_{0}$ (kPa) | $E_{\infty}$(kPa) | *τ* (s) | $E_{1}$(kPa) | *α* |
| Force curves | 2.3±0.1 | 1.63±0.02 | 0.044±0.07 | 2.3±1.6 | 0.08±0.02 |
| Stress relaxation | 1.9±0.1 | 1.6±0.1 | 0.056±0.002 | 2.3±1.6 | 0.05±0.01 |
| Microrheology* | 2.2±0.1 | 1.6±0.1 | 0.06±0.01 | 2.2±1.4 | 0.08±0.02 |

Viscoelastic properties of cells have been investigated by AFM using several different experimental setups. In the time domain, creep and stress relaxation experiments have been conducted in a large number of studies. Short (64 ms^25^), medium (2 s^26^), and long (120 s^27^) creep/relaxation times have been used, but thermal drift in the instrument can become an issue at longer times^28^. Difficulties in interpretation may also arise from active cell responses to the applied forces, and active processes may dominate over passive relaxation^29^. Generally, Heaviside step-loading was assumed before the hold phase (step-hold experiments)^19,30,31^. However, in an actual experiment, maximal loading speed is limited by hydrodynamic and inertial effects and step-loading conditions are impossible to implement. The fit of the total ramp-hold curve^32^ or introduction of the ramp correction factor should be implemented for proper analysis^33,34^. Both SLS and PLR models have been used for the data description in step-hold experiments with cells, with direct comparisons made in some of the studies^33,35^. However, no consensus about the model prevalence was observed. Other models include the generalized Maxwell model with two^26,33^ and three relaxation times ^30^, the stretched exponential function^36^, poroelasticity model^31,37^ and fractional variations of the SLS model^38^.

Experiments in the frequency domain are also popular. Here the cantilever is sinusoidally oscillated with fixed small amplitude and several frequencies during the period of indentation^20,21,39–43^, or during the scanning process^44,45^. Frequency-dependent complex Young’s or shear modulus is measured from the amplitude and phase shift of the cantilever displacement. A power-law structural-damping model, which is an analog of the PLR model in the frequency domain, described reasonably well data obtained on cells for frequencies below 100-200 Hz^20,41,46^. Because such measurements take place during the period of indentation (generally about 500 nm depth^20,47^), the ongoing global cell relaxation process could add complexity to the analysis. If a prolonged lag time is added before the beginning of oscillations, the active cell response could affect the measurements.

Several authors attempted to assess viscoelastic properties directly from force curves. Approach-retraction hysteresis can provide such parameters as energy loss^48–50^, the apparent viscosity of sample^51^, and the difference between calculated apparent modulus for approach and retraction curves^52^. However, these parameters are mostly not based on a complete viscoelastic solution like the Ting’s model and are thus defined as “apparent”. Indentation-rate dependency of apparent elastic modulus was used to obtain the power law exponent^8,50^ or relaxation time^53^ of cells, but such a method requires the acquisition of several force curves at different piezo-displacement speeds.

The main advantage of the method described here is that it has a firm theoretical foundation for viscoelastic analysis – Ting’s model – and can be applied directly to conventional AFM force curves. Therefore, it shares the limitations and uncertainties of the usual AFM experiments, including cantilever stiffness and optical lever sensitivity calibration^54^. Other limitations are absence of strong adhesion forces during the retraction; drifts and active cell response at low indentation speeds; relatively high computational costs. The method could be applied to the most indentation experiments, where indentation and force histories are available, conducted not only with AFM. All indentation data points are used for processing and no lag times before measurements are required. Measurement time may be adjusted by varying the piezo speed. Different viscoelastic relaxation models can be used and compared. The method may further benefit from instruments with direct measurement of cantilever deflection and velocity as in laser Doppler vibrometry. Overall, the methods presented here enhance the potential of AFM for use in cell mechanics assays.

1. **Additional data for the hydrogels**

All measurements on polyacrylamide (PAAm) hydrogels were performed in PBS buffer containing up to 0.1% Triton X-100 detergent to decrease probe-gel adhesion. Adhesion forces could be caused by interactions between negatively charged colloidal probe and local charges presented along the polyacrylamide chains; also, disentanglement of chains upon retraction could occur (observed as multiple unstable rupture events upon separation)^12,55^. An example of force curve with high adhesion is shown in Fig. S9A. The large adhesive forces are responsible for the prominent part of the approach-retraction hysteresis and thus make viscoelastic analysis inapplicable. It was shown before^12,56^ that surfactants, detergents or blocking agents like BSA (bovine serum albumin) could be used to decrease the adhesion. Here we used a non-ionic surfactant Triton X-100, which dramatically reduced probe-hydrogel adhesion forces (Fig. S9B).


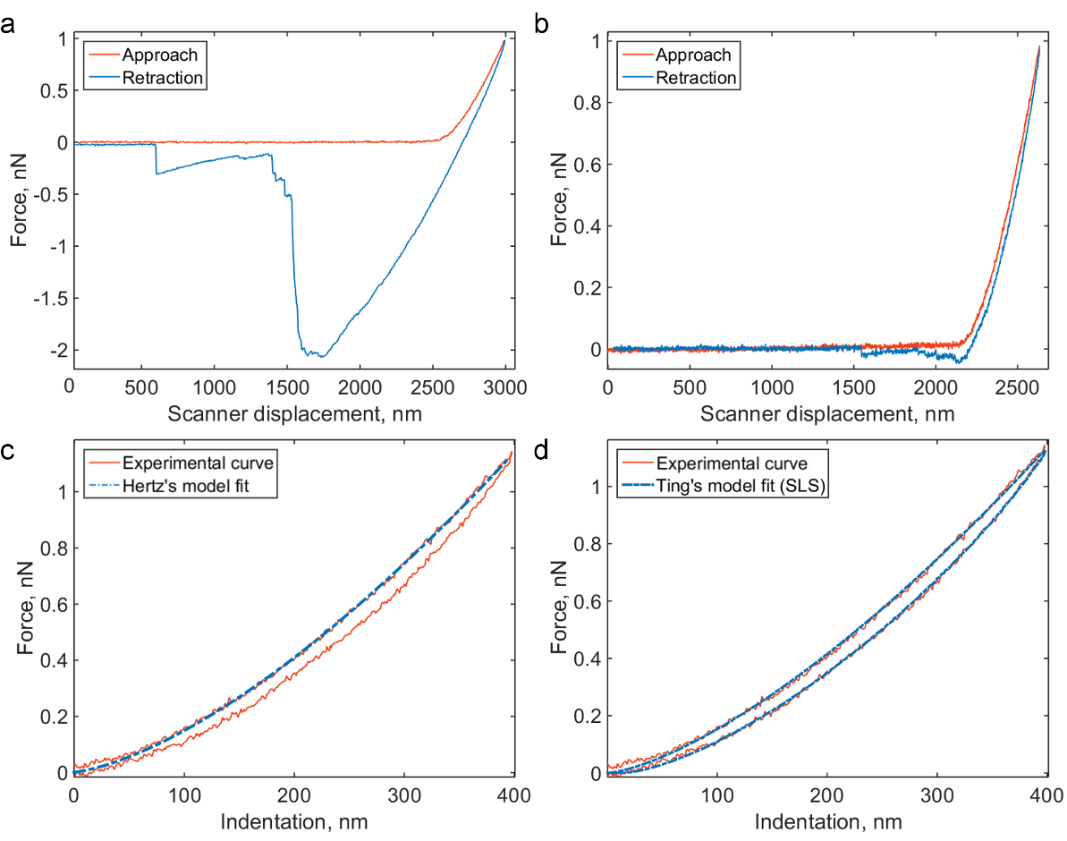


**Figure S9. Reduction of adhesion in force curves obtained on PAAm hydrogels after addition of Triton X-100. a,** An example of strong adhesive interactions. **b,** Reduced adhesion after addition of 0.1% Triton X-100. **c,** *F-δ* curve (2 μm/s piezo speed) fitted with the Hertz’s model. **d,** The same curve with the Ting’s (SLS) model fit.

Three PAAm hydrogels samples were analysed. The SLS model was applied for the processing of the data (Fig. S9). The apparent Young’s modulus $E_{Hertz}$ was 1.7 ± 0.1 kPa (at 2 μm/s scanner displacement speed); $E_{0}$ = 2.3 ± 0.2 kPa; $E_{\infty}$ = 1.63±0.02 kPa; relaxation time *τ* = 0.044±0.07 s. Since the relaxation time was much lower than the indentation time (about 0.4 s for 2 μm/s scanner displacement speed), $E_{Hertz}$ values were close to $E_{\infty}$ values. Th*e* $E_{\infty}/E_{0}$ ratio was ~0.71 indicating moderate degree of viscoelastic behaviour.

To check the assumption about linear viscoelastic behaviour we varied the indentation depth in the range 100-1,200 nm. The *τ* values decreased in the region of low (<300nm) indentations, probably due to the effects of long-range forces and contact point uncertainties, but stabilized at higher indentations (Fig. S10). Weak growth in the $E_{Hertz}$, $E_{0}$ and $E_{\infty}$ moduli values was also observed.


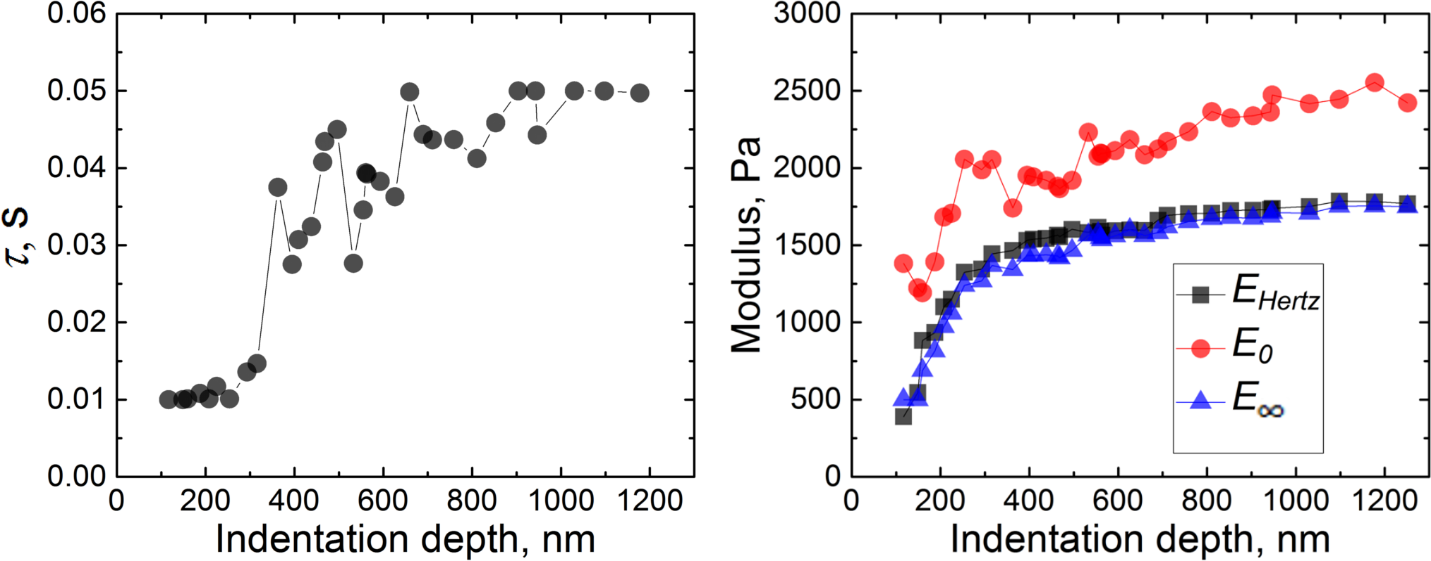


**Figure S10. Plots of *τ*,** $E_{Hertz}$**,** $E_{0}$ **and** $E_{\infty}$ **as a function of indentation depth for PAAm hydrogel*.***

In experiments with the varied piezo speed the $E_{0}$ and $E_{\infty}$ moduli values were almost constant, while $E_{Hertz}$ demonstrated a growth from $E_{\infty}$ toward $E_{0}$. Such behaviour is expected for the SLS model (Fig. S11A). In the case of NIH 3T3 cells, where the PLR model was applied, $E_{Hertz}$ increased following a weak power law behaviour while $E_{0}$ values did not change significantly (Fig. S11B). We also calculated the normalized hysteresis area as the area enclosed between the approach and retraction curves divided by the area under the approach curve. The normalized hysteresis area was remarkably small (almost 0) for PAAm hydrogels at piezo speeds below 0.5 μm/s and then increased with the increasing piezo speed. For NIH 3T3 cells, the normalized hysteresis area was much less dependent on the piezo speed, demonstrating only a slight increase as predicted by PLR model (Fig. S11C).


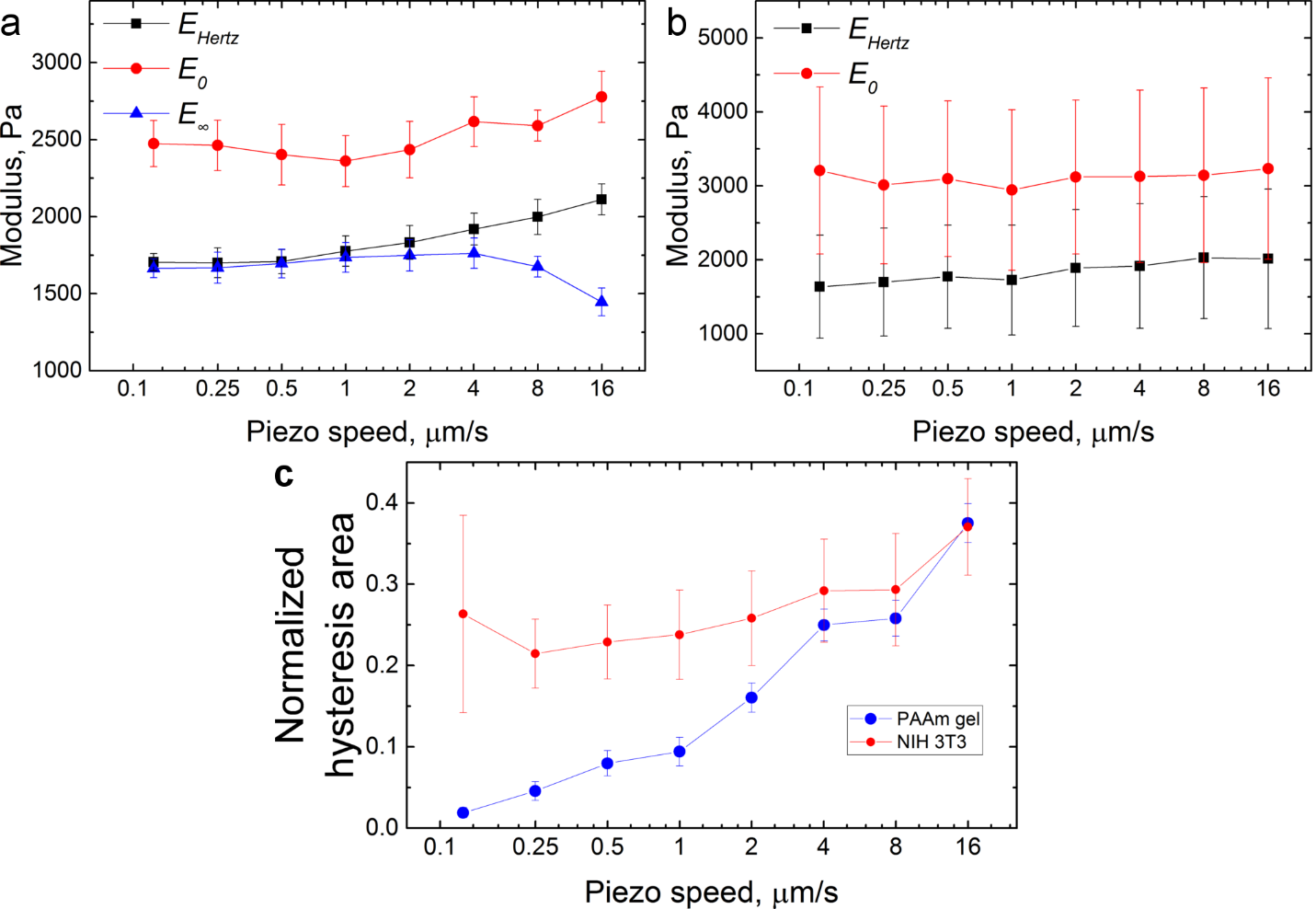


**Figure S11. Plots of** $E_{Hertz}$**,** $E_{0}$**,** $E_{\infty}$**, and normalized hysteresis area as a function of piezo displacement speed for PAAm hydrogel and NIH 3T3 cells*.*** **a,** $E_{Hertz}$, $E_{0}$ and $E_{\infty}$ for PAAm hydrogel, SLS model was applied for the processing of data. **b,** $E_{Hertz}$ and $E_{0}$ for NIH 3T3 cells, PLR model was applied for the processing of data. **c,** Normalized hysteresis area for both PAAm hydrogel and NIH 3T3 cells.

1. **Additional data for the studied cell lines**

Distributions of $E_{Hertz}$, $E_{0}$, and *α* for the cell lines NIH 3T3, NMuMG, MDA-MB-231, and MCF-7 are shown in Fig. S12. The benign cell lines NIH 3T3 and NMuMG had higher values of $E_{0}$ and lower values of *α* than the cancer cells MDA-MB-231 and MCF-7 (p<0.01 between all parameters except $E_{0}$ for the pair of cancer cells and *α* for the pair of benign cells). However, we did not see the correlation between $E_{0}$ and *α* inside the single cell line data (Fig. S13A), while apparent Young’s modulus measured with the Hertz’s model $E_{Hertz}$ and *α* demonstrated weak negative correlation (Pearson's r from -0.1 for NIH 3T3 to -0.6 for MDA-MB-231, p<0.05), larger for cancerous cells (Fig. S13B). A strong significant negative correlation was observed between *α* and $E_{Hertz}/E_{0}$ ratio (≈-0.9, p<0.001), meaning that $E_{Hertz}$ values depend strongly on the amount of relaxation (Fig. S13C). $E_{Hertz}/E_{0}$ratio versus *α* dependence were described quite closely (R^2^ = 0.94) with exponential decay function $\frac{E_{Hertz}}{E_{0}}=e^{\left(\frac{-\alpha}{0.173}\right)}$ (Fig. S13C).


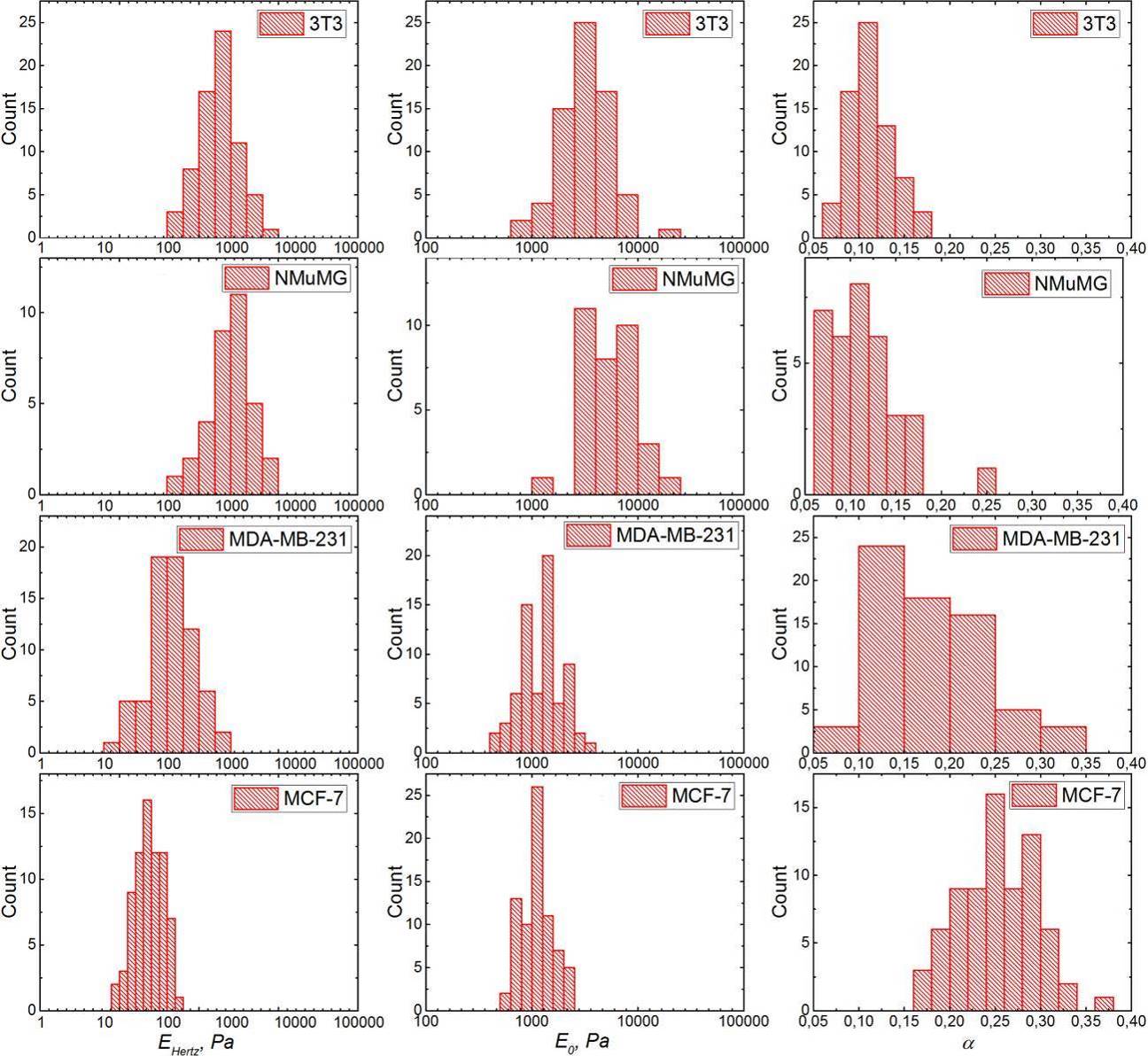


**Figure S12. Distributions of** $E_{Hertz}$**,** $E_{0}$**, and *α* for the studied cell lines.**

**
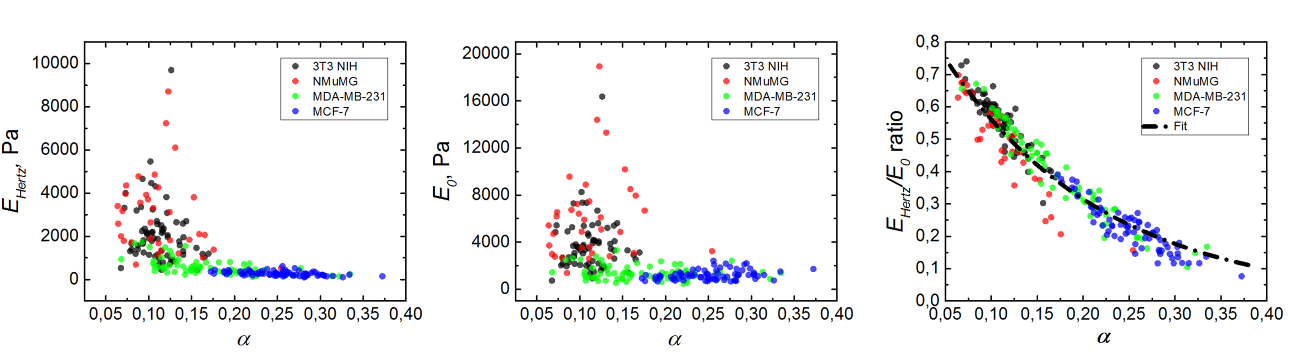
**

**Figure S13. Plots of** $E_{Hertz}$**,** $E_{0}$**, and** $E_{Hertz}/E_{0}$ **ratio as a function of *α* for the studied cell lines*.***

We used the developed technique to compare the difference between control and Syk-expressing MDA-MB-231 cells^57^. The Syk protein-tyrosine kinase is supposed to play roles in tumour progression, acting as an inhibitor of cellular motility and metastasis in highly invasive cancer cells. A line of MDA-MB-231 cells expressing Syk-EGFP upon incubation with tetracycline analog was used^58^, the expression of Syk-EGFP was induced by the addition of 1 μg/mL doxycycline for 24 h, and only cells with green fluorescence were analysed. Both an increase in $E_{0}$ (by ≈20%, p =0.02) as well as a decrease in *α* (by ≈14%, p =0.01) in Syk-expressing cells were observed, which together lead to an even higher increase in $E_{Hertz}$ (≈34%, p <0.01) (Table 2 in the main text, Fig. S14).


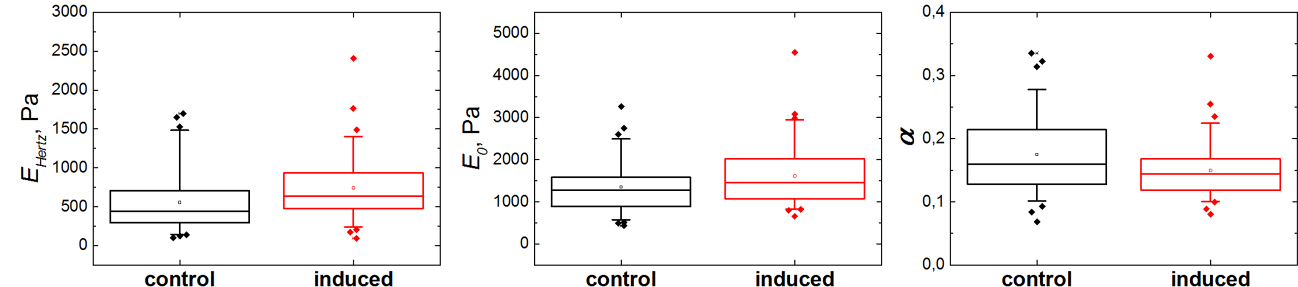


**Figure S14. Viscoelastic parameters of MDA-MB-231 cells without (control) and with Syk-EGFP expression (induced).** Box plots of apparent Young’s modulus $E_{Hertz}$, short-term modulus $E_{0}$ and power-law exponent *α*. Difference between all distribution is significant at the p<0.01 level***.***

We also investigated changes in mechanical properties of NMuMG cells during epithelial-to-mesenchymal transition (EMT). Previous research with a AFM microrheology technique shown that *α* values decrease after TGF-β-induced EMT^42^. The same trend was observed here. After 96h of induction with 10 ng/mL of TGF-β, the cells went from epithelial (monolayer) to mesenchymal-like state and had viscoelastic parameters close to that of single untreated cells, which also have a mesenchymal-like morphology (Fig. S15, Tables 1 and 3 in the main text). The $E_{0}$ value was approximately preserved during the transition (nonsignificant difference between all groups), while *α* values decreased almost two-fold and became close to that of single untreated cells. This resulted in approximately the same difference in the $E_{Hertz}$ values. Thus, after transition cells became more solid-like, apparently due to the assembly of the stress fibres and increased pre-stress stored in the membrane and actin cortex^42^.


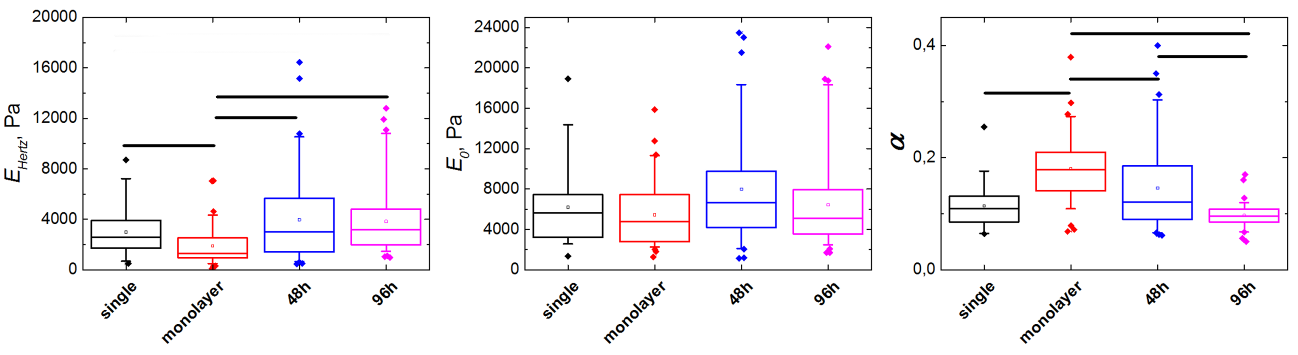


**Figure S15. Viscoelastic parameters of NMuMG cells before and during EMT (48h and 96h induction with TGF-β).** Box plots of apparent Young’s modulus $E_{Hertz}$ short-term modulus $E_{0}$ and power-law exponent *α*. Difference between marked groups is significant at the level p<0.001.

Variation in the indentation depth on the same cell in the range 200-1,000 nm did not lead to significant changes in $E_{Hertz}$, $E_{0}$, and *α*, except larger values and scatter were obtained for low (<200nm) indentations, probably caused by long-range forces and other uncertainties in this region (Fig. S16).


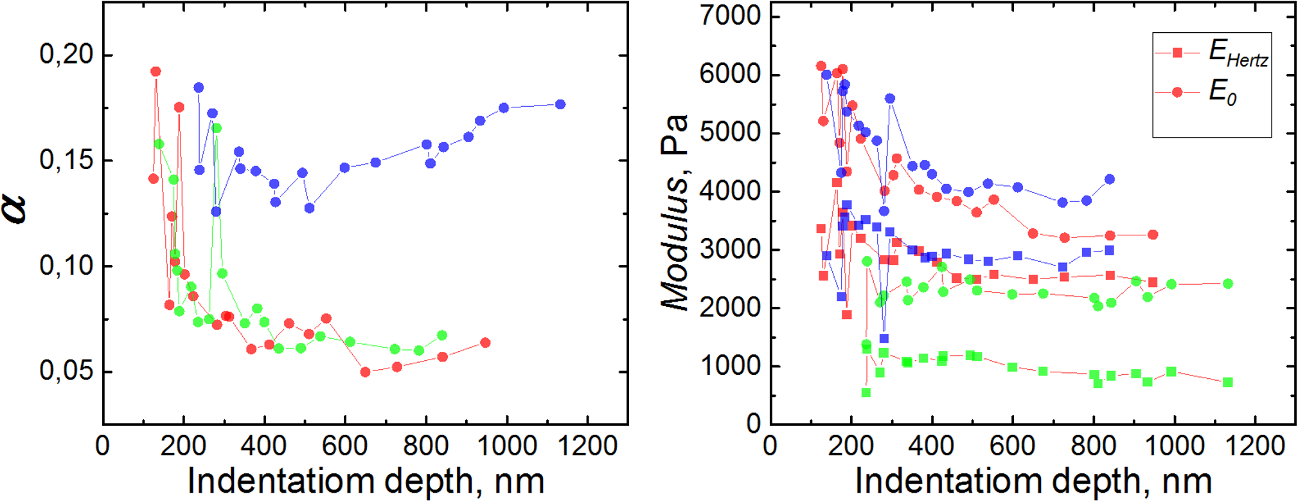


**Figure S16. Plots of *α*,** $E_{Hertz}$**, and $E_{0}$ as a function of indentation depth for three NIH 3T3 cells (different colours indicate different experiment cells)*.***

1. **Additional information for the developed method**

We checked the possibility of extracting viscoelastic parameters using only the data from the approach phase of *F-δ* curves. In this case, the simpler Lee-Radok solution can be used^59^. However, this approach gave results that were inconsistent with those of the complete Ting’s model-based analysis. When reconstructed with Ting’s model, *F-δ* curves followed experimental curves closely in the approach phase, but failed in the description of the retraction phase (data not shown). The reason for this observed behaviour again seemed to lie in uncertainties in the beginning of the indentation region. When only the approach part was analysed, these uncertainties hampered the true relaxation behaviour and led to wrong relaxation estimations.

It was suggested that Lee-Radok model is approximately valid for the beginning part of the retraction phase in several previous works^60,61^. Here we also checked this proposition by processing approach together with first half of retraction curve (for force values larger than 50% of its peak value) with Lee-Radok solution. Overall, this model provides close fits of the experimental curves but tends to underestimate both $E_{0}$ and *α* by 10-15% relative to the complete Ting’s analysis results. It should be noted that Lee-Radok solution-based algorithm is faster than Ting’s solution-based algorithm, because there is no need to calculate $t_{1}\left(t\right)$ function, and could be used to promptly assess the cell viscoelastic properties or to obtain initial guesses for the complete Ting’s analysis.

Although it is tempting to make an assumption about constant indentation speed, considering it to be equal to the rate of the piezo extension, $d\delta\left(t\right)/dt \approx dz\left(t\right)/dt$, this may introduce a significant error in the results even for soft samples like cells. Such an assumption may greatly simplify the analysis, and an analytical solution might be obtained for some viscoelastic models for the hereditary integrals in Lee-Radok model equation. But, as seen in Fig. S2D and Fig. S4F, the actual indentation speed is not constant during the experiment, it decreases with the indentation depth because of the cantilever deflection. Implementation of the constant indentation speed in the Ting’s algorithm provided $E_{0}$ and *α* values underestimated by 10-20%, and even higher (by 50%) for the stiffest cells where the cantilever deflects stronger per same indentation depth (data not shown).

**References**

1. Ting, T. C. T. The contact stresses between a rigid indenter and a viscoelastic half-space. *J. Appl. Mech.* **33,** 845–854 (1966).

2. Graham, G. A. C. The contact problem in the linear theory of viscoelasticity when the time dependent contact area has any number of maxima and minima. *Int. J. Eng. Sci.* **5,** 495–514 (1967).

3. Hunter, S. C. The Hertz problem for a rigid spherical indenter and a viscoelastic half-space. *J. Mech. Phys. Solids* **8,** 219–234 (1960).

4. Bilodeau, G. G. Regular Pyramid Punch Problem. *J. Appl. Mech.* **59,** 519–523 (1992).

5. Niu, T. & Cao, G. Finite size effect does not depend on the loading history in soft matter indentation. *J. Phys. D. Appl. Phys.* **47,** 385303 (2014).

6. Dimitriadis, E. K., Horkay, F., Maresca, J., Kachar, B. & Chadwick, R. S. Determination of elastic moduli of thin layers of soft material using the atomic force microscope. *Biophys. J.* **82,** 2798–2810 (2002).

7. Gavara, N. & Chadwick, R. S. Determination of the elastic moduli of thin samples and adherent cells using conical atomic force microscope tips. *Nat. Nanotechnol.* **7,** 733–736 (2012).

8. Efremov, Y. M. *et al.* The effects of confluency on cell mechanical properties. *J. Biomech.* **46,** 1081–1087 (2013).

9. Méndez-Méndez, J. V, Alonso-Rasgado, M. T., Correia Faria, E., Flores-Johnson, E. A. & Snook, R. D. Numerical study of the hydrodynamic drag force in atomic force microscopy measurements undertaken in fluids. *Micron* **66,** 37–46 (2014).

10. Alcaraz, J. *et al.* Correction of microrheological measurements of soft samples with atomic force microscopy for the hydrodynamic drag on the cantilever. *Langmuir* **18,** 716–721 (2002).

11. Johnson, K. & Greenwood, J. An adhesion map for the contact of elastic spheres. *J. Colloid Interface Sci.* **192,** 326–333 (1997).

12. Efremov, Y. M., Bagrov, D. V., Kirpichnikov, M. P. & Shaitan, K. V. Application of the Johnson–Kendall–Roberts model in AFM-based mechanical measurements on cells and gel. *Colloids Surfaces B Biointerfaces* **134,** 131–139 (2015).

13. Lin, Y. Y., Hui, C. Y. & Baney, J. M. Viscoelastic contract, work of adhesion and the JKR technique. *J. Phys. D. Appl. Phys.* **32,** 2250–2260 (1999).

14. Attard, P. Measurement and interpretation of elastic and viscoelastic properties with the atomic force microscope. *J. Phys. Condens. Matter* **19,** 473201 (2007).

15. Johnson, K. L. Contact mechanics and adhesion of viscoelastic spheres. *Microstruct. Microtribology Polym. Surfaces* **741,** 24–41 (1999).

16. Rutland, M., Tyrrell, J. & Attard, P. Analysis of atomic force microscopy data for deformable materials. *J. Adhes. Sci. Technol.* **18,** 1199–1215 (2004).

17. Hiratsuka, S. *et al.* Power-Law Stress and Creep Relaxations of Single Cells Measured by Colloidal Probe Atomic Force Microscopy. *Jpn. J. Appl. Phys.* **48,** 08JB17 (2009).

18. Niu, T. & Cao, G. Power-law rheology characterization of biological cell properties under AFM indentation measurement. *RSC Adv.* **4,** 29291–29299 (2014).

19. Darling, E., Zauscher, S. & Guilak, F. Viscoelastic properties of zonal articular chondrocytes measured by atomic force microscopy. *Osteoarthritis Cartilage* **14,** 571–579 (2006).

20. Alcaraz, J. *et al.* Microrheology of human lung epithelial cells measured by atomic force microscopy. *Biophys. J.* **84,** 2071–2079 (2003).

21. Efremov, Y. M. *et al.* Distinct impact of targeted actin cytoskeleton reorganization on mechanical properties of normal and malignant cells. *Biochim. Biophys. Acta* **1853,** 3117–3125 (2015).

22. Lakes, R. S. *Viscoelastic Solids*. (CRC Press, 1998).

23. Fabry, B. *et al.* Scaling the Microrheology of Living Cells. *Phys. Rev. Lett.* **87,** 1–4 (2001).

24. Smith, B. A., Tolloczko, B., Martin, J. G. & Grütter, P. Probing the viscoelastic behavior of cultured airway smooth muscle cells with atomic force microscopy: stiffening induced by contractile agonist. *Biophys. J.* **88,** 2994–3007 (2005).

25. Hecht, F. M. *et al.* Imaging viscoelastic properties of live cells by AFM: power-law rheology on the nanoscale. *Soft Matter* **11,** 4584–4591 (2015).

26. Moreno-Flores, S., Benitez, R., Vivanco, M. D. & Toca-Herrera, J. L. Stress relaxation microscopy: imaging local stress in cells. *J. Biomech.* **43,** 349–354 (2010).

27. Stamenović, D. *et al.* Rheological behavior of living cells is timescale-dependent. *Biophys. J.* **93,** L39–L41 (2007).

28. Weafer, P. P. *et al.* Stability enhancement of an atomic force microscope for long-term force measurement including cantilever modification for whole cell deformation. *Rev. Sci. Instrum.* **83,** 93709 (2012).

29. Jonas, O. & Duschl, C. Force propagation and force generation in cells. *Cytoskeleton* **67,** 555–563 (2010).

30. Babahosseini, H., Carmichael, B., Strobl, J. S., Mahmoodi, S. N. & Agah, M. Sub-cellular force microscopy in single normal and cancer cells. *Biochem. Biophys. Res. Commun.* **463,** 587–592 (2015).

31. Moeendarbary, E. *et al.* The cytoplasm of living cells behaves as a poroelastic material. *Nat. Mater.* **12,** 253–61 (2013).

32. Tripathy, S. & Berger, E. J. Measuring viscoelasticity of soft samples using atomic force microscopy. *J. Biomech. Eng.* **131,** 94507 (2009).

33. Wang, B. *et al.* A general approach for the microrheology of cancer cells by atomic force microscopy. *Micron* **44,** 287–297 (2013).

34. Qiang, B., Greenleaf, J., Oyen, M. & Zhang, X. Estimating material elasticity by spherical indentation load-relaxation tests on viscoelastic samples of finite thickness. *IEEE Trans. Ultrason. Ferroelectr. Freq. Control* **58,** 1418–1429 (2011).

35. Hemmer, J. D. *et al.* Role of Cytoskeletal Components in Stress-Relaxation Behavior of Adherent Vascular Smooth Muscle Cells. *J. Biomech. Eng.* **131,** 41001 (2009).

36. Okajima, T. *et al.* Stress relaxation of HepG2 cells measured by atomic force microscopy. *Nanotechnology* **18,** 84010 (2007).

37. Galli, M., Comley, K. S. C., Shean, T. A. V. & Oyen, M. L. Viscoelastic and poroelastic mechanical characterization of hydrated gels. *J. Mater. Res.* **24,** 973–979 (2009).

38. Carmichael, B., Babahosseini, H., Mahmoodi, S. N. & Agah, M. The fractional viscoelastic response of human breast tissue cells. *Phys. Biol.* **12,** 46001 (2015).

39. Takahashi, R. & Okajima, T. Mapping power-law rheology of living cells using multi-frequency force modulation atomic force microscopy. *Appl. Phys. Lett.* **107,** 173702 (2015).

40. Dokukin, M. & Sokolov, I. High-resolution high-speed dynamic mechanical spectroscopy of cells and other soft materials with the help of atomic force microscopy. *Sci. Rep.* **5,** 12630 (2015).

41. Rother, J., Nöding, H., Mey, I. & Janshoff, A. Atomic force microscopy-based microrheology reveals significant differences in the viscoelastic response between malign and benign cell lines. *Open Biol.* **4,** 140046 (2014).

42. Schneider, D. *et al.* Tension monitoring during epithelial-to-mesenchymal transition links the switch of phenotype to expression of moesin and cadherins in NMuMG cells. *PLoS One* **8,** e80068 (2013).

43. Rebelo, L. M., Sousa, J. S. De, Santiago, T. M. & Filho, J. M. in *Microscopy: advances in scientific research and education* (ed. Méndez-Vilas, A.) 141–152 (Formatex Research Center, 2014).

44. Cartagena-Rivera, A. X., Wang, W.-H., Geahlen, R. L. & Raman, A. Fast, multi-frequency, and quantitative nanomechanical mapping of live cells using the atomic force microscope. *Sci. Rep.* **5,** 11692 (2015).

45. Raman, a. *et al.* Mapping nanomechanical properties of live cells using multi-harmonic atomic force microscopy. *Nat. Nanotechnol.* **6,** 809–814 (2011).

46. Hiratsuka, S. *et al.* The number distribution of complex shear modulus of single cells measured by atomic force microscopy. *Ultramicroscopy* **109,** 937–941 (2009).

47. Roca-Cusachs, P. *et al.* Rheology of passive and adhesion-activated neutrophils probed by atomic force microscopy. *Biophys. J.* **91,** 3508–3518 (2006).

48. Collinsworth, A. M., Zhang, S., Kraus, W. E. & Truskey, G. A. Apparent elastic modulus and hysteresis of skeletal muscle cells throughout differentiation. *Am. J. Physiol. Physiol.* **283,** C1219–C1227 (2002).

49. Mathur, A. B., Collinsworth, A. M., Reichert, W. M., Kraus, W. E. & Truskey, G. A. Endothelial, cardiac muscle and skeletal muscle exhibit different viscous and elastic properties as determined by atomic force microscopy. *J. Biomech.* **34,** 1545–1553 (2001).

50. Nawaz, S. *et al.* Cell Visco-Elasticity Measured with AFM and Optical Trapping at Sub-Micrometer Deformations. *PLoS One* **7,** e45297 (2012).

51. Rebelo, L. M., de Sousa, J. S., Mendes Filho, J. & Radmacher, M. Comparison of the viscoelastic properties of cells from different kidney cancer phenotypes measured with atomic force microscopy. *Nanotechnology* **24,** 55102 (2013).

52. Prabhune, M., Belge, G., Dotzauer, A., Bullerdiek, J. & Radmacher, M. Comparison of mechanical properties of normal and malignant thyroid cells. *Micron* **43,** 1267–1272 (2012).

53. Caporizzo, M. A. *et al.* Strain-rate Dependence of Elastic Modulus Reveals Silver Nanoparticle Induced Cytotoxicity. *Nanobiomedicine* **2,** 9 (2015).

54. Wagner, R., Moon, R., Pratt, J., Shaw, G. & Raman, A. Uncertainty quantification in nanomechanical measurements using the atomic force microscope. *Nanotechnology* **22,** 455703 (2011).

55. Nalam, P. C., Gosvami, N. N., Caporizzo, M. a, Composto, R. J. & Carpick, R. W. Nano-rheology of hydrogels using direct drive force modulation atomic force microscopy. *Soft Matter* (2015). doi:10.1039/C5SM01143D

56. Ebenstein, D. M. Nano-JKR force curve method overcomes challenges of surface detection and adhesion for nanoindentation of a compliant polymer in air and water. *J. Mater. Res.* **26,** 1026–1035 (2011).

57. Krisenko, M. O., Cartagena, A., Raman, A. & Geahlen, R. L. Nanomechanical Property Maps of Breast Cancer Cells As Determined by Multiharmonic Atomic Force Microscopy Reveal Syk-Dependent Changes in Microtubule Stability Mediated by MAP1B. *Biochemistry* **54,** 60–68 (2014).

58. Zhang, X., Shrikhande, U., Alicie, B. M., Zhou, Q. & Geahlen, R. L. Role of the protein tyrosine kinase Syk in regulating cell-cell adhesion and motility in breast cancer cells. *Mol. Cancer Res.* **7,** 634–644 (2009).

59. Lee, E. H. & Radok, J. R. M. The Contact Problem for Viscoelastic Bodies. *J. Appl. Mech.* **27,** 438–444 (1960).

60. Peng, G., Zhang, T., Feng, Y. & Huan, Y. Determination of shear creep compliance of linear viscoelastic-plastic solids by instrumented indentation when the contact area has a single maximum. *Polym. Test.* **31,** 1038–1044 (2012).

61. Vandamme, M. & Ulm, F. J. Viscoelastic solutions for conical indentation. *Int. J. Solids Struct.* **43,** 3142–3165 (2006).

**Supplementary Movies**

**
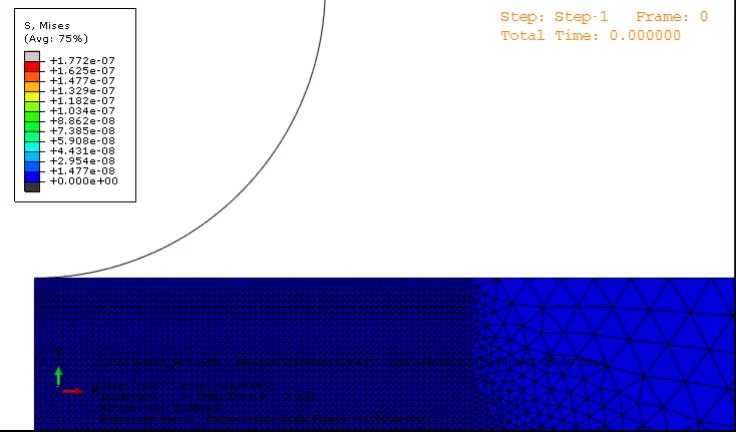
**

**Movie S1. FE simulation of the indentation process for the material described with PLR model. The distribution of effective von Mises stress is presented.**
